# A meta-analysis on *Dirofilaria immitis* and *Dirofilaria repens* in countries of North Africa and the Middle East

**DOI:** 10.1017/S003118202500037X

**Published:** 2025-04

**Authors:** Katie Izenour, Fayez Salib, Jordan Eckert, Jeba R.J. Jesudoss Chelladurai, Lindsay Starkey, Byron Blagburn, Christine Sundermann, Janna Willoughby, Sarah Zohdy

**Affiliations:** 1 Department of Pathobiology, Auburn University, Auburn, AL, USA; 2 Faculty of Veterinary Medicine, Cairo University, Giza Governorate, Cairo Egypt; 3 Department of Mathematics and Statistics, Auburn University, Auburn, AL, USA; 4 Department of Veterinary Pathobiology, Oklahoma State University, Stillwater, OK, USA; 5 Department of Biological Sciences, Auburn University, Auburn, AL, USA; 6 College of Forestry, Wildlife, and Environment, Auburn University, Auburn, AL, USA

**Keywords:** *Dirofilaria immitis*, *Dirofilaria repens*, global animal health, heartworm, meta-analysis, Middle East, North Africa, veterinary medicine

## Abstract

*Dirofilaria immitis* and *D. repens* are globally distributed mosquito-borne parasitic filarial nematodes. Data on the prevalence of *Dirofilaria* spp. is not aggregated or publicly available at the national level for countries in North Africa and the Middle East. A systematic review and meta-analysis of publications describing cases of *D. immitis* and *D. repens* in 21 countries in North Africa and the Middle East was performed following PRISMA guidelines to estimate the prevalence of *Dirofilaria* spp. where national and regional estimates don’t exist. In total, 460 publications were reviewed, and 34 met all inclusion criteria for the meta-analysis model. This analysis found that the combined prevalence of *Dirofilaria* spp. in the included countries was 2.4% (95% CI: 1.6–3.6%; *I*^2^ = 81.7%, 95% CI: 78.6–84.3%). Moderator analysis showed a statistically significant difference in the prevalence estimate between diagnostic test methods used. The model detected a high degree of heterogeneity among studies and publication bias. Removal of model identified outliers reduced the estimated prevalence from 2.4% to 1.0%, whereas the trim-and-fill analysis suggested a higher adjusted prevalence (12%). Despite these findings, *Dirofilaria* spp. prevalence is likely dynamic due to seasonal variations in mosquito vector populations and differences in local mosquito control practices. Additional studies from the countries in and surrounding this region are needed to better identify key risk factors for *Dirofilaria* spp. in domestic canids and other species (including humans) to inform prevention and control decisions to limit further transmission.

## Graphical abstract

**Figure:**
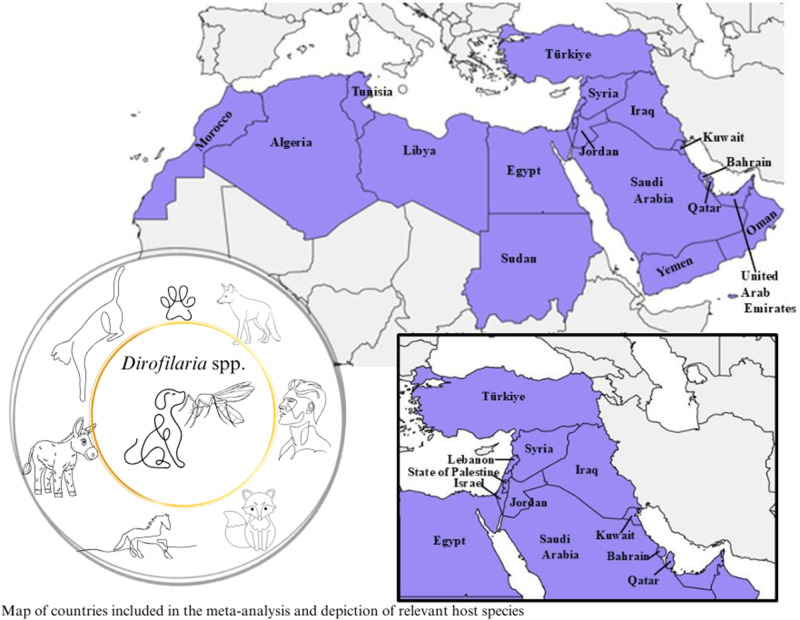

## Introduction

*Dirofilaria immitis* and *D. repens* are parasitic, filarial nematodes of epidemiological importance in both human and veterinary medicine (Genchi and Kramer, 2020). *D. immitis* causes canine heartworm disease in dogs; other species can be accidental hosts such as felids (Villanueva-Saz et al., 2021; Fagundes-Moreira et al., 2024), jackals and foxes (Otranto et al., 2019; Potkonjak et al., 2020). *Dirofilaria repens* causes subcutaneous dirofilariasis (Genchi and Kramer, 2017) in numerous mammalian species including canids such as red foxes, golden jackal and wolves (Rishniw et al., 2006; Capelli et al., 2018; Potkonjak et al., 2020). Nodules from *D. repens* can be located in the subcutaneous tissue, conjunctiva or thoracic wall (Choudhury et al., 2023) of an infected host.

Mosquitoes serve as the intermediate host for both *D. immitis* and *D. repens*. The overlapping presence of competent mosquito vectors and *Dirofilaria* spp. infected dogs in the right climactic conditions are required for transmission to occur. Both species are zoonotic, known to cause pulmonary (Simón et al., 2003; Kozlov et al., 2023; Tsai et al., 2024), subcutaneous (Popescu et al., 2012; Falidas et al., 2016) and ocular lesions (Aykur et al., 2021; Redón-Soriano et al., 2022) in humans (Simón et al., 2012); additional public health impact on humans remains unclear. There are several diagnostic methods that can be used depending on the host and presentation of symptoms. Across host species, identification of microfilaria in the peripheral blood (Ciuca et al., 2020) or recovery of adult worms from pleural fluid (Valčiukaitė-Žilinskienė et al., 2024), pulmonary artery (Gregory et al., 2023) or a skin lesion (Falidas et al., 2016) are the most common methods for diagnosis of both parasites.

**Figure 1.**
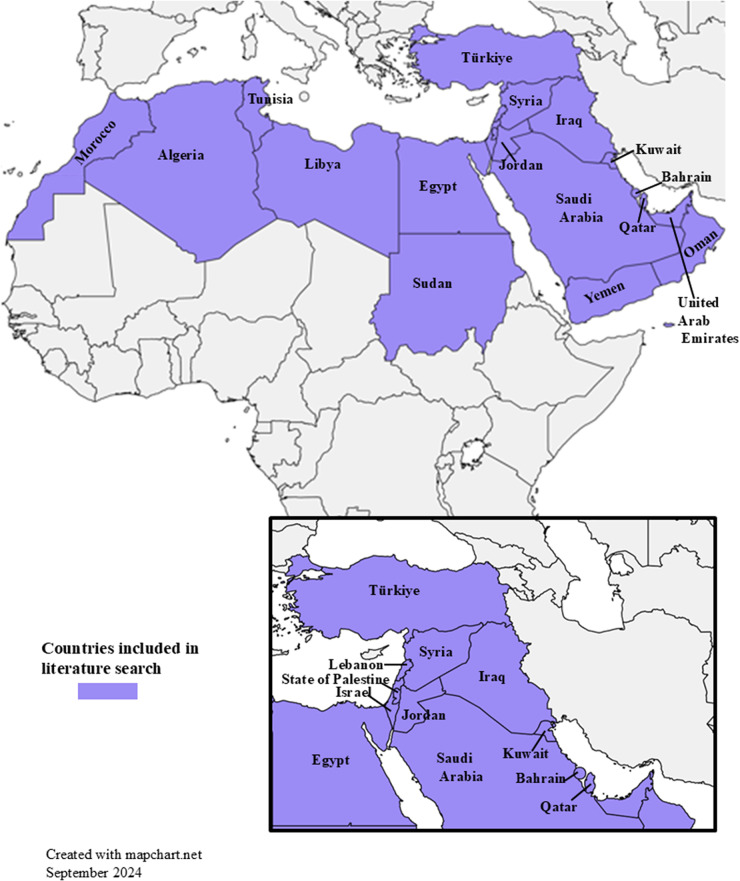
Map highlighting all 21 countries included in the literature search. The map inset is a zoomed in view of some of the countries in the Middle East along the Mediterranean Sea.

It is expected that the distribution and prevalence of *D. immitis* and *D. repens* follow similar patterns to the distribution of dogs infected with *Dirofilaria* (McKay et al., 2013; Alberigi et al., 2023) and competent mosquito vectors of *D. immitis* and *D. repens* belonging to the genera *Aedes, Anopheles* and *Culex* (Dyab et al., 2015). Therefore, prevalence of *D. immitis* and *D. repens* is expected in countries in North Africa and the Middle East. Specifically, competent mosquito vectors are known to be present, such as, *Culex* and *Aedes* in Türkiye (Biskin et al., 2010) and *Aedes, Anopheles* and *Culex* in Egypt (Dyab et al., 2015) as well as dogs infected with *Dirofilaria* spp. (Baneth et al., 2002; Selim et al., 2021); however, details of specific mosquito vectors are lacking. The climate of regions in North Africa and surrounding the Mediterranean (not to be confused with WHO MENA region), including Algeria, Bahrain, Egypt, Iraq, Israel, Jordan, Kuwait, Lebanon, Libya, Mauritania, Morocco, Oman, Occupied Palestinian Territory (State of Palestine), Qatar, Saudi Arabia, Sudan, Syria, Tunisia, Türkiye (Turkey), the United Arab Emirates and Yemen (Figure 1), are conducive to development and transmission of *Dirofilaria* spp. (Bowman and Atkins, 2009; Bowman and Wu, 2022; Atkinson et al., 2024). This is an area where pathogen transmission would be expected because of the overlap of host, vector and suitable climate. These 21 counties are significant and similar in the Mediterranean and North Africa because they have arid desert climates (Varela et al., 2020) and share social and cultural norms that may impact companion animal ownership (Mohr et al., 2017; Mohamed et al., 2019).

To date, there is no publicly available surveillance or reporting system for *Dirofilaria* spp. in any of the countries in North Africa, and the Middle East. Therefore, published reports are the only publicly accessible sources of information describing the location, host/vector species, diagnostic test used and prevalence of these 2 pathogens in the countries in this area. The data in these publications can be used to estimate pooled prevalence in a meta-analysis (Barendregt et al., 2013). The goal of this meta-analysis is to estimate the prevalence and geographic distribution of *Dirofilaria* spp., specifically *D. immitis* and/or *D. repens*, in the countries listed above where no national prevalence estimates are available. This analysis is the first to estimate the prevalence of *Dirofilaria* spp. in these countries and can be used by veterinarians and public health policy makers to guide animal owner education and outreach programmes and inform medical doctors about the zoonotic risk of these pathogens.

Additionally, this meta-analysis describes factors (moderators) that impact the prevalence of *D. immitis* and *D. repens* such as diagnostic approach and test used, *Dirofilaria* species detected, host species sampled and continent of origin. Publication bias is also examined. While other studies have described different aspects of the prevalence of *D. immitis* and *D. repens*, these authors are not aware of other meta-analyses estimating the prevalence of these parasites in the countries of interest (Table 1).

**Table 1.** Prior systematic reviews and meta-analyses of *D. immitis* and *D. repens* describing North Africa and the Middle East

Title and citation	Year
‘Heartworm adulticide treatment: a tropical perspective’ (Dantas-Torres et al., 2023)	2023
‘Prevalence of *Dirofilaria immitis* in mosquitoes (Diptera) – systematic review and meta-analysis’ (Riahi et al., 2021)	2021
‘The global status of *Dirofilaria immitis* in dogs: a systematic review and meta-analysis based on published articles’ (Anvari et al., 2020)	2020
‘The prevalence of *Dirofilaria immitis* and *D. repens* in the Old World’ (Genchi and Kramer, 2020)	2020
‘What is happening outside North America regarding human dirofilariasis?’ (Simon et al., 2005)	2005

## Methods

### Literature search and data filtering

**Figure 2.**
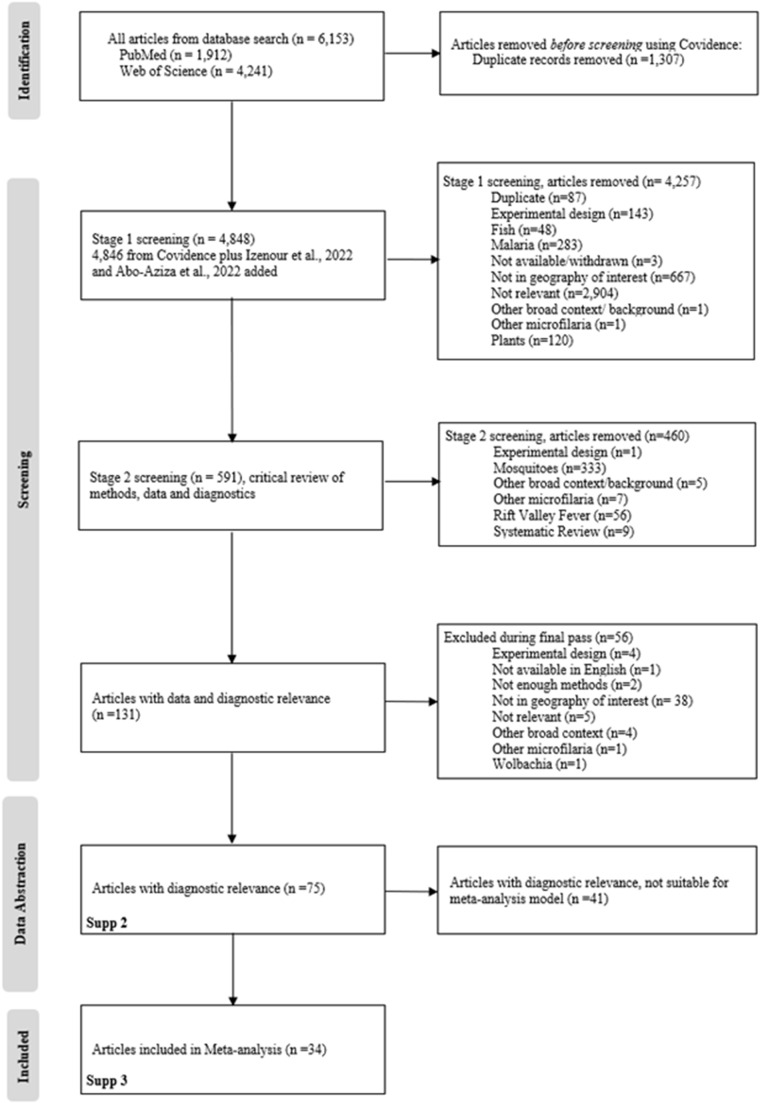
PRISMA flow diagram, depicting the publication identification, review and inclusion process for the systematic review and meta-analysis. It includes the number of publications excluded at each step and the reason for exclusion.

This literature review and meta-analysis follows the 2020 The Preferred Reporting Items for Systematic reviews and Meta-Analyses (PRISMA) checklist and flow diagram guidance (Page et al., 2021) (Figure 2). The literature search was conducted in April 2022 from the English language databases, PubMed (National Institute of Health. National Center for Biotechnology Information. U.S. National Library of Medicine, 2022) and Clarivate™ (Web of Science™ Core Collection) for publications reporting the identification and/or detection of ‘*D. immitis*’, ‘*D. repens*’, ‘*Dirofilaria immitis*’, ‘*Dirofilaria repens*’ or ‘heartworm’ in any of the following countries or territories: ‘Morocco’, ‘State of Palestine’, ‘Occupied Palestinian Territory’, ‘Tunisia’, ‘Algeria’, ‘Egypt’, ‘Libya’, ‘Mauritania’, ‘Sudan’, ‘Oman’, ‘Saudi Arabia’, ‘Yemen’, ‘Iraq’, ‘Jordan’, ‘Syria’, ‘Lebanon’, ‘Israel’, ‘Kuwait’, ‘Qatar’, ‘the United Arab Emirates’, ‘the United Arab Emirate’, ‘Bahrain’, ‘Türkiye’ or ‘Turkey’ (Supplement 1). Results included all the relevant literature from 1986 to 2022. The search did not have limitations on host species, or diagnostic or detection method.

Covidence (Veritas Health Innovation, Melbourne, Australia) was used to manage the database of identified publications. First, author(s) name and publication title were assessed to identify and remove duplicate records. From the remaining publications, any that did not contain diagnostic information on *D. immitis* or *D. repens* in known and relevant hosts or locations of interest were removed. Other reasons for publication exclusion include reports of fish or plants, or reports of cases from countries not included in the search query list. Systematic reviews, review articles and other meta-analyses were removed because no new case data were contained in them. The remaining 591 publications proceeded to an in-depth assessment to identify those with the relevant pathogen detection data in the geographic range targeted by these analyses. At this stage, publications were excluded if they reported travel-related cases; only reported detection of other species of filaria, such as *Wuchereria bancrofti* (Moustafa et al., 2017; Dahesh and Ibrahim, 2018); those that experimentally infected animals; and publications about Rift Valley Fever (removed 460 publications). Finally, an additional intensive review of the remaining publications, focused on diagnostic methods and origin of infection. In this stage, publications with insufficient description of diagnostic method(s) and, the geographic origin of the patient or their infection was not clearly described were excluded.

The remaining 75 publications (Supplement 2) fully met all inclusion/exclusion criteria (referred to as ‘Full dataset’ from here on). From the ‘Full dataset’ of 75 publications, a subset of 34 publications (Supplement 3) was created for the meta-regression model (referred to as ‘Meta-analysis dataset’ from here on). This selection was based on satisfying inclusion criteria for the model to be properly powered and bias minimized; specifically, the publications included in the ‘Meta-analysis dataset’ have a sample size (denominator) greater than 3.

### Data preparation

Data extracted from each of the ‘Full dataset’ publications to create the ‘Meta-analysis dataset’ included information about the source such as country of infection, city/region/province of infection, species sampled (i.e. dog, cat, human, and mosquito) and sample type (i.e. blood, serum, worms and mosquitoes). Other extracted data included total sample size, total number positive for *D. immitis* or *D. repens, Dirofilaria* species detected, and diagnostic test/technique used. Data from each publication were extracted by a single researcher into a Microsoft® Excel® (version 2408) spreadsheet as separate observations.

The meta-analysis dataset, model and analysis were structured and performed following the established methods and best practices (Harrer et al., 2022). Across the entire ‘Meta-analysis dataset’, an individual moderator (such as host species) must occur thrice for there to be enough weight in the analysis and satisfy data requirements for model performance. Observations from publications that otherwise met the inclusion criteria but occurred less than thrice had to be excluded from the model (Harrer et al., 2019). One example of this is in Otranto et al. (2019), in which the authors reported PCR results from fox and jackal host species. No other publications reported testing on these host species, so they could not be included in the ‘Meta-analysis dataset’, but these observations are retained in the ‘Full dataset’. Other exceptions included publications from Tunisia and Jordan remained in the model even though they contribute 1 publication each with less than 3 rows of data because these locations were underrepresented in the dataset. Repeated testing on the same animal (e.g. a rapid test and PCR performed on blood from the same animal) was treated as independent tests and each sample/test combination was recorded as separate observations in the ‘Meta-analysis dataset’ because the results of one test did not influence administration of the other. A similar approach was used when a diagnostic test was performed on a subset of the original sample. The original sample and subset were treated as 2 separate samples, for example from the whole population, the ones that tested positive using the first diagnostic method then underwent a second diagnostic method.

### Meta-analysis

Meta-analysis using R (R version 4.3.3, R Foundation for Statistical Computing, Vienna Austria, 2021) with the following statistical packages was performed: *readxl* version 1.4.3 (Wickham and Bryan, 2023), *metasens* version 1.5-2 (Schwarzer et al., 2023), *metafor* version 4.4-0 (Viechtbauer, 2010), *meta* version 7.0-0 (Balduzzi et al., 2019), *tidyverse* version 2.0.0 (Wickham et al., 2019), *devtools* version 2.4.5 (Wickham et al., 2022) and *dmetar* version 0.1.0 (Harrer et al., 2019) (R code in Supplement 4). A mixed-effects model, *k* = 132 events (also called observations), estimated overall prevalence was fit from 34 publications. Forest plots for the diagnostic test method aggregate moderator visualized the overlap of each observations’ confidence interval. Outlier analysis was performed to identify observations with confidence intervals outside the 95% confidence limit of the pooled effect (Harrer et al., 2019). Influence analysis was performed using the ‘Leave-One-Out’ paradigm internally (Harrer et al., 2019) to produce a Baujat plot, which evaluates the relationship between heterogeneity and influence of each event.

Moderator analysis was guided by identification of common data themes in the included publications and consultations with *Dirofilaria* subject matter experts. The moderators are diagnostic method, diagnostic method type (aggregation of diagnostic method), *Dirofilaria* species, host species and continent of origin. Finally, to explore sources of publication bias, a contour-enhanced funnel plot of all observations in the dataset was created. Trim-and-fill analysis was conducted on both the complete ‘Meta-analysis dataset’ (*k* = 132 observations, where an observation is 1 independent prevalence data point extracted from each publication) and on the dataset with the identified outliers removed.

## Results

### Description of relevant publications

This literature search followed PRISMA (Page et al., 2021) (Figure 2) guidelines, for publication selection. The search in PubMed returned 1912 publications and Web of Science returned 4241, totaling 6153. Covidence (Veritas Health Innovation, Melbourne, Australia) was used to remove duplicates, leaving 4846 publications (1307 removed). The original literature search was conducted in 2022; in 2024, the search was conducted again to identify any new publications, and this identified 2 additional publications (Izenour et al., 2022; Abo-Aziza et al., 2022), bringing the total number of records reviewed to 4848. The first stage of filtering identified publications that reported new data on *Dirofilaria* spp. in the targeted geographic region and resulted in the removal of 4257 publications. Of the 591 publications remaining, misalignment of topics in the publication with the stated research goals resulted in the removal of an additional 460 publications. Another 56 publications were removed because there was insufficient description of the diagnostic method. This left 75 publications (Supplement 2) with diagnostic data that fully met all inclusion/exclusion criteria (‘Full dataset’).

### Description of ‘Full dataset’

The ‘Full dataset’ (Supplement 2) includes publications from 11 countries: Algeria (*n* = 2), Egypt (*n* = 7), Iraq (*n* = 2), Israel (*n* = 9), Jordan (*n* = 1), Kuwait (*n* = 3), Morocco (*n* = 2), Saudi Arabia (*n* = 4), Tunisia (*n* = 10), Türkiye (*n* = 33) and the United Arab Emirates (*n* = 2). These publications reported sampling these host species: humans, dogs, cats, donkeys, horses, jackals, mosquitoes and foxes. *D. immitis* was reported in all host species except horses, donkeys and foxes (Table 2). *D. repens* was reported in all host species except jackals, horses and foxes. Publications from 7 countries: Egypt (Abdel-Rahman et al., 2008), Israel (Munichor et al., 2001; Raniel et al., 2006), Kuwait (Hira et al., 1994), Saudi Arabia (Chopra et al., 2004), Tunisia (Sassi et al., 2006; Fleck et al., 2009), Türkiye (Koltas et al., 2002; Beden et al., 2007) and the United Arab Emirates (Mittal et al., 2008) reported human cases of *D. repens*; 2 countries, Tunisia (Ziadi et al., 2005) and Türkiye (Aykur et al., 2021), reported human cases of *D. immitis* (Table 3). The ‘Full dataset’ did not meet model criteria, and the pooled prevalence was not calculated.

**Table 2.** All reports of *D. immitis* or *D. repens* in any host or vector species by country

Country	Species of *Dirofilaria*	Host positive for *Dirofilaria* spp.	Vector positive for *Dirofilaria* spp.	Vector species by country
Algeria	*D. immitis*	Dog (Meriem-Hind and Mohamed, 2009; Tahir et al., 2017)		*Aedes albopictus*
				*Culex pipiens*
				*Culiseta longiareolata*
				*Culex quinquefasciatus* (Abdellahoum et al., 2022)
Bahrain	Not reported			*Anopheles* spp. (Ismaeel et al., 2004)
Egypt	*D. immitis*	Cat (Al-Kappany et al., 2011)	*Culex* spp. (Dyab et al., 2015)	*Anopheles* spp. (Dyab et al., 2015)
		Dog (Selim et al., 2021)		
	*D. repens*	Dog (Abdullah et al., 2021)	*Aedes* spp. (Dyab et al., 2015)	*Aedes aegypti* (Abozeid et al., 2018)
		Donkey (Abo-Aziza et al., 2022)	*Culex* spp. (Dyab et al., 2015)	
		Human (Abdel-Rahman et al., 2008)		
Iraq	*D. immitis*	Dog (Otranto et al., 2019; Tarish et al., 1986)		*Culex* spp. (Sharifi et al., 2022)
		Jackal (Otranto et al., 2019)		
	*D. repens*	Dog (Otranto et al., 2019)		
Israel	*D. repens*	Dog (Baneth et al., 2002; Harrus et al., 1999; Mazaki-Tovi et al., 2016)		*Aedes* spp. *Culex* spp. (Abbasi et al., 2022; Behar et al., 2021)
		Human (Chazan et al., 2001; Govrin-Yehudain et al., 2017; Gutierrez et al., 1995; Munichor et al., 2001; Raniel et al., 2006; Stayerman et al., 1999)		
Jordan	*D. immitis*	Dog (Obaidat and Alshehabat, 2018)		*Aedes* spp. (Kanani et al., 2017)
				*Culex* spp. (Al-Tammemi and Shtaiyat, 2024)
Kuwait	*D. repens*	Dog (Tarello, 2008)		*Culex* spp. (Colton et al., 2019)
		Human (Hira et al., 2008; Hira et al., 1994)		*Culiseta* spp. (Reeves et al., 2016)
Lebanon	Not reported			*Aedes* spp. (Haddad et al., 2022)
				*Culex* spp.
				*Culiseta* spp. (Zakhia et al., 2021)
Libya	Not reported			*Aedes* spp.
				*Anopheles* spp.
				*Culex* spp. (Nebbak et al., 2022)
Mauritania	Not reported			*Aedes vexans*
				*Culex poicilipes*
				*Culex antennatus* (Barry et al., 2022; El Ghassem et al., 2023)
Morocco	*D. immitis*	Dog (Elhamiani Khatat et al., 2017; Pandey et al., 1987)		*Anopheles laranchae* (Faraj et al., 2008)
				*Aedes* spp. (Abdelkrim et al., 2024)
				*Culex pipiens* (Arich et al., 2024; Arich et al., 2022)
				*Culiseta* spp. (Nebbak et al., 2022)
Oman	Not reported			*Aedes aegypti* (Al-Abri et al., 2020)
				*Anopheles* spp.
				*Culex* spp. (Ullah et al., 2020)
Occupied Palestinian Territory/State of Palestine	Not reported			*Aedes* spp. (Allah Adawi, 2012)
Qatar	Not reported			*Culex* spp.
				*Aedes* spp.
				*Anopheles* spp. (Tahir et al., 2022)
Saudi Arabia	*D. immitis*	Cat (Omar et al., 2018)		*Anopheles* spp. (Ahmed et al., 2011)
		Dog (Omar et al., 2018; Tarello, 2003)		*Aedes* spp. (Ahmed et al., 2011)
	*D. repens*	Dog (Tarello, 2003)		*Culex* spp. (Ahmed et al., 2011; Alahmed et al., 2019)
		Human (Chopra et al., 2004; Dababo et al., 2022)		
Sudan	Not reported			*Anopheles* spp. (Abdelwhab et al., 2021)
				*Aedes* spp. (Ali El Hadi Mohamed et al., 2020)
				*Culex* spp. (Ali El Hadi Mohamed et al., 2020)
Syria	Not reported			*Aedes* spp. (Haddad et al., 2007)
Tunisia	*D. immitis*	Dog (Chabchoub et al., 2003; Rjeibi et al., 2017)		*Aedes* spp. (Ben Ayed et al., 2019; Bohers et al., 2020)
		Human (Ziadi et al., 2005)		
	*D. repens*	Dog (Rjeibi et al., 2017)		*Anopheles* spp. (Tabbabi and Daaboub, 2017; Tabbabi and Daaboub, 2018; Tabbabi et al., 2018)
		Human (Fleck et al., 2009; Kaouech et al., 2010; Makni et al., 2007; Mrad et al., 1999; Saied et al., 2011; Soussi et al., 2004; Ziadi et al., 2005)		*Culex* spp. (Tabbabi and Daaboub, 2017; Tabbabi and Daaboub, 2018; Tabbabi et al., 2018)
Türkiye	*D. immitis*	Dog (Adanir et al., 2013; Atas et al., 2018; Ceribasi and Simsek, 2012; Cetinkaya et al., 2016; Ceylan et al., 2021; Colak et al., 2020; Guven et al., 2017; Icen et al., 2011; Köse and Erdogan, 2012; Oge et al., 2003; Oncel and Vural, 2005; Pasa et al., 2017; Sevimli et al., 2007; Simsek and Ciftci, 2016; Simsek et al., 2008; Tasci and Kilic, 2012; Ural et al., 2014; Voyvoda et al., 2004; Yaman et al., 2009; Yildirim et al., 2007; Yildiz et al., 2008)	*Aedes* spp. (Biskin et al., 2010; Demirci et al., 2021; Yildirim et al., 2011)	
			*Anopheles* spp. (Demirci et al., 2021)	
			*Culex* spp. (Demirci et al., 2021)	
		Human (Aykur et al., 2021)		
	*D. repens*	Dog (Simsek and Ciftci, 2016)	*Culex* spp.	
		Human (Beden et al., 2007; Koltas et al., 2002; Kutluturk et al., 2016; Latifoglu et al., 2002)	*Aedes* spp.	
			*Anopheles* spp. (Demirci et al., 2021)	
	*D. repens* and *D. immitis* co-infection	Dog (Simsek and Ciftci, 2016)	*Culex* spp.	
			*Aedes* spp.	
			*Anopheles* spp. (Demirci et al., 2021)	
United Arab Emirates	*D. repens*	Dog (Tarello, 2002), Human (Mittal et al., 2008)		*Aedes* spp.
				*Anopheles* spp.
				*Culex* spp. (Camp et al., 2019)
Yemen	Not reported			*Aedes* spp. (Tucker et al., 2020)
				*Anopheles* spp. (Assada et al., 2024)

**Table 3.** Human cases of *Dirofilaria* spp. by country

Country	*Dirofilaria* spp.	Sample source	Total sample	Total positive	Publication (author last name year)
Egypt	*D. repens*	Worm and blood	3	3	(Abdel-Rahman et al., 2008)
Israel	*D. repens*	Worms	1	1	(Chazan et al., 2001)
		Worms	1	1	(Munichor et al., 2001)
		Worms	1	1	(Raniel et al., 2006)
		Worms	1	1	(Govrin-Yehudain et al., 2017)
		Worms	1	1	(Gutierrez et al., 1995)
		Worms	1	1	(Stayerman et al., 1999)
Kuwait	*D. repens*	Worms	1	1	(Hira et al., 2008)
		Worms	1	1	(Hira et al., 1994)
Saudi Arabia	*D. repens*	Worms	1	1	(Chopra et al., 2004)
		Worms	1	1	(Dababo et al., 2022)
Tunisia	*D. immitis*	Worms	1	1	(Ziadi et al., 2005)
	*D. repens*	Worms	1	1	
		Worms	1	1	(Fleck et al., 2009)
		Worms	1	1	(Saied et al., 2011)
		Worms	1	1	(Mrad et al., 1999)
		Worms	1	1	(Sassi et al., 2006)
		Worms	1	1	(Soussi et al., 2004)
		Worms	1	1	(Makni et al., 2007)
		Worms	1	1	(Kaouech et al., 2010)
Turkey	*D. immitis*	Worms	1	1	(Aykur et al., 2021)
	*D. repens*	Worms	1	1	(Beden et al., 2007)
		Worms	1	1	(Koltas et al., 2002)
		Worms	3	2	(Kutluturk et al., 2016)
		Worms	1	1	(Latifoglu et al., 2002)
United Arab Emirates	*D. repens*	Worms	1	1	(Mittal et al., 2008)

### Description of publications for meta-analysis model

The ‘Meta-analysis dataset’ (Supplement 3), a subset of the ‘Full dataset,’ was used to calculate the pooled prevalence. It contains data extracted from the final 34 publications that met all inclusion criteria and had sufficient data for the meta-analysis model (denominator >3 and more than 3 occurrences of a variable across the entire dataset). These publications ranged in publication date from 1986 to 2022 from 10 countries: Algeria (*n* = 2), Egypt (*n* = 4), Iraq (*n* = 1), Israel (*n* = 1), Jordan (*n* = 1), Kuwait (n = 1), Morocco (*n* = 2), Saudi Arabia (*n* = 1), Tunisia (*n* = 1) and Türkiye (*n* = 20). The publications in the ‘Meta-analysis dataset’ reported 14 different diagnostic testing methods, all publications used blood or serum for the test sample and all sampled only dogs or cats as host species. *D. immitis* was the most frequently detected parasite, reported in 7 of 10 countries represented in the data and 29 of 34 publications included for meta-analysis. *D. repens* was reported in 6 of 10 countries and 6 of 34 publications.

### Meta-analysis model

The mixed-effects model using the 34 publications (*k* = 132 observations, where an observation is 1 prevalence data point extracted from each publication) in the ‘Meta-analysis dataset’ estimated the combined prevalence of *D. immitis* and *D. repens* for all countries, diagnostic methods and species to be 2.4% (95% CI: 1.6–3.6%; *I*^2^ = 81.7%, 95% CI of *I*^2^ = 78.6–84.3%). Outlier analysis identified 39 observations as outliers. All of the outlier observations reported positive detection of *Dirofilaria* spp. These outliers came from publications from Algeria (*n* = 2; 5%), Israel (*n* = 1; 3%), Morocco (*n* = 3; 7%), Saudi Arabia (*n* = 2; 5%), Tunisia (n = 1; 3%) and Türkiye (*n* = 30; 77%). Most outlier observations reported a rapid test type (69%). Microscopy was the next most common (23%), followed by PCR (8%). The model removed these 39 observations, leaving 93 observations. The estimated prevalence of *D. immitis* and *D. repens* with outliers removed was 1.0% (95% CI: 0.71–1.5%; *I*^2^ = 0.0%, 95% CI = 0.0–25.2%).

### Exploration of heterogeneity

**Figure 3.**
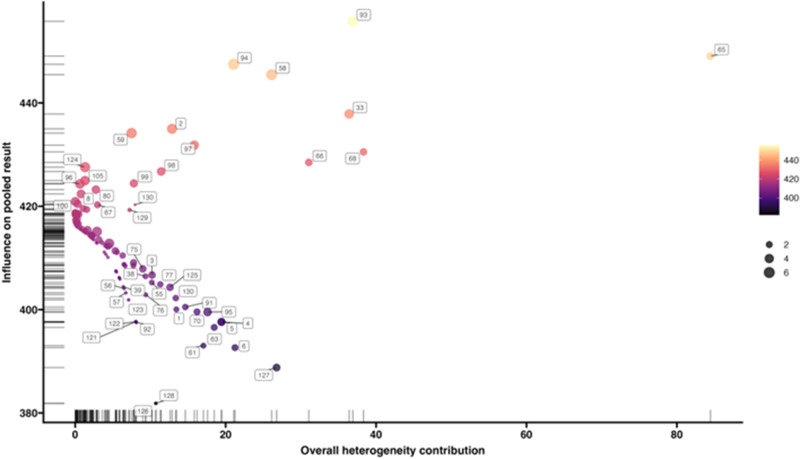
Baujat plot of the meta-analysis dataset. It displays each observation’s contribution of heterogeneity along the horizontal axis and the influence of the pooled result along the vertical axis. Observations with more heterogeneity or influence can be visually detected with this plot.

The Baujat plot (Figure 3) of the mixed-effects model for all observations in the ‘Meta-analysis dataset’ visualizes the relationship between heterogeneity and influence of each observation. Observations 65, 68, 33 and 93 have the highest heterogeneity. Observations 93, 94, 58 and 33 have the highest influence.

### Effects of continent of origin on prevalence

Publications in the ‘Meta-analysis dataset’ came from countries in 2 continents – Asia (*n*=108 observations) and Africa (n = 24 observations) (Supplement 5A). The estimated prevalence of *D. immitis* and *D. repens* in Asian countries in this dataset was 2.5% (95% CI: 1.6–3.9%; *I*^2^ = 81.0%) and in African countries in this dataset was 2.0% (95% CI: 0.7–5.2%; *I*^2^ = 84.4%). There was not a statistically significant difference in prevalence estimates between continents.

### Effect of host species on prevalence

Diagnostic samples in the ‘Meta-analysis dataset’ were derived from 2 host species – dogs (*n* = 127) and cats (*n* = 5) (Supplement 5B). The estimated prevalence of *Dirofilaria* spp. among dogs was 2.6% (95% CI: 1.7–3.9%; *I*^2^ = 81.7%) and among cats was 0.48% (95% CI: 0.02–11.5%; *I*^2^ = 0.0%). There was not a statistically significant difference in prevalence between the 2 host species.

### Effect of pathogen species on prevalence

*D. immitis* was tested in 121 observations in the ‘Meta-analysis dataset’, whereas *D. repens* was tested in 11 observations (Supplement 5C). The mixed-effects model estimated the prevalence of *D. immitis* to be 2.7% (95% CI: 1.8–4.0%; *I*^2^ = 81.0%), and the prevalence of *D. repens* to be 0.9% (95% CI: 0.05–14.3%; *I*^2^ = 55.1%). There was no statistically significant difference between species of parasite detected.

### Effect of diagnostic tests on prevalence

The mixed-effects model of the diagnostic tests moderator was first conducted on the 14 individual diagnostic tests reported (Supplement 5D). The most commonly reported diagnostic test, DiroCHEK^®^ Canine Heartworm Antigen Test Kit (Zoetis, Florham Park, NJ), *n* = 31 publications, estimated the prevalence of *Dirofilaria* spp. to be 6.5% (95% CI: 4.0–10.5%; *I*^2^ = 88%) and contributed the most heterogeneity, *I*^2^ = 88%. The lowest prevalence was estimated from observations that used the Knotts method, 0.3% (95% CI: 0.0–100.0%; *I*^2^ = 0.0%). Prevalence was estimated to be <1% for observations that used each of the following 4 test methods: Knotts (0.3%), Microscopy (Giemsa stain) (0.5%), PCR (0.7%) and Microscopy blood smear (0.8%).

Some of the diagnostic tests were reported fewer than 3 times, such as the FilarCHECK Ag ELISA (Agrolabo, Scarmagno, Italy), IDEXX SNAP^®^ 4Dx^®^ Test (IDEXX, Westbrook, ME) and the SNAP^®^ Feline Triple^®^ (IDEXX, Westbrook, ME), so a second moderator analysis was performed with the diagnostic tests aggregated by test type to increase the number of observations. The 14 diagnostic tests could be classified into 3 groupings: (1) Rapid test to detect antigen (8 different antigen rapid tests reported), (2) ‘Microscopy’ to detect microfilaria of *Dirofilaria* spp. or adults of *D. repens* (5 different microscopy methods reported) and (3) PCR to detect *Dirofilaria* spp. DNA (Supplement 5E). The estimated prevalence of both parasites varied based on diagnostic test group used: 3.9% (95% CI: 2.4–6.3%; *I*^2^ = 82.3%) for the grouped rapid antigen tests, 2.3% (95% CI: 0.98–5.2%; *I*^2^ = 79.5%) for the grouped microscopy techniques and 0.7% (95% CI: 0.2–2.4%; *I*^2^ = 81.1%) for PCR. There was a statistically significant difference in prevalence between at least 2 of the aggregated test types. However, this test does not indicate which ones, it only indicates that there is a difference between at least 2 of the tests. Diagnostic test type was the only moderator with a statistically significant influence on prevalence. Moderator analysis of continent of origin, host species and pathogen species did not produce statistically significant results.

**Figure 4.**
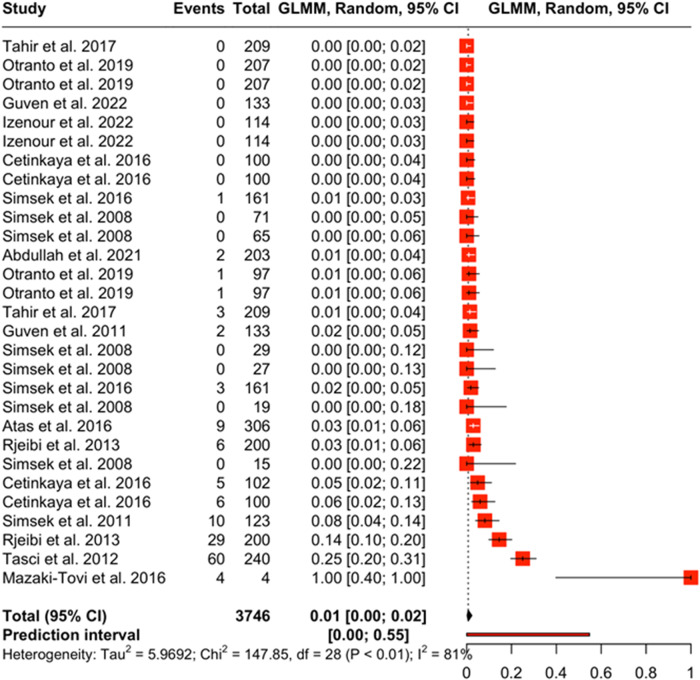
The forest plot shows the effect size of each observation against a predicted effect size (diamond symbol). This forest plot is from the PCR diagnostic method moderator analysis.

**Figure 5.**
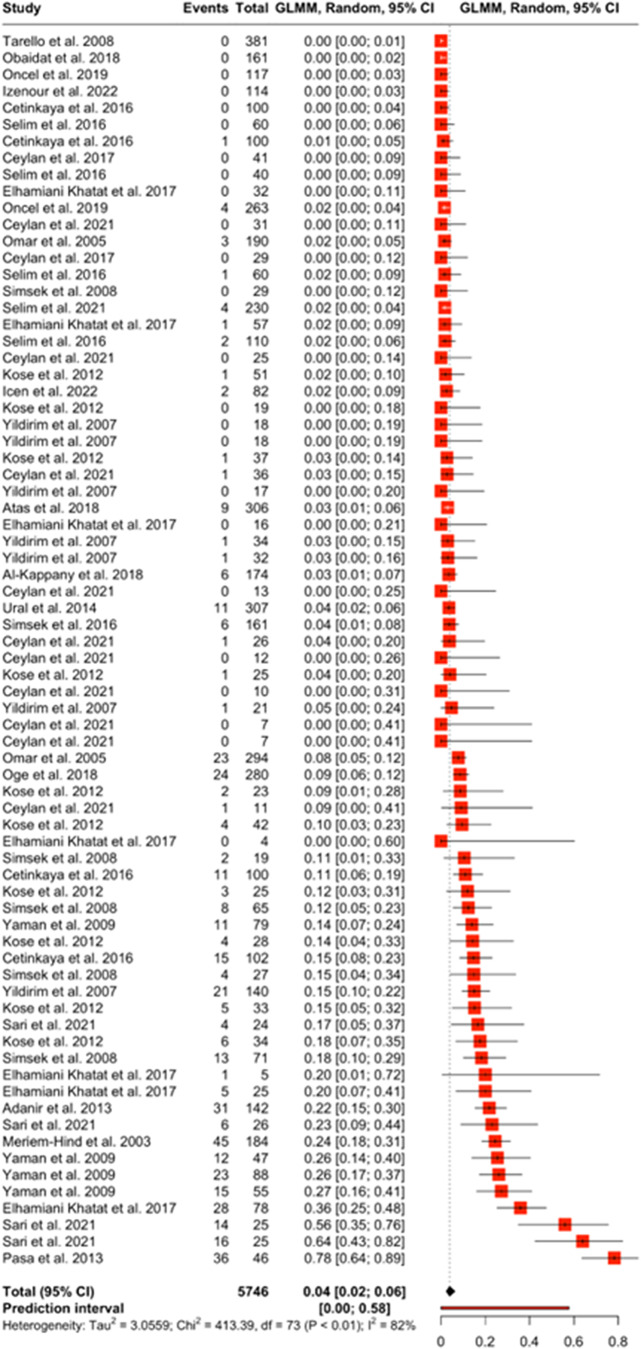
The forest plot shows the effect size of each observation against a predicted effect size (diamond symbol). This forest plot is from the rapid test diagnostic method moderator analysis.

**Figure 6.**
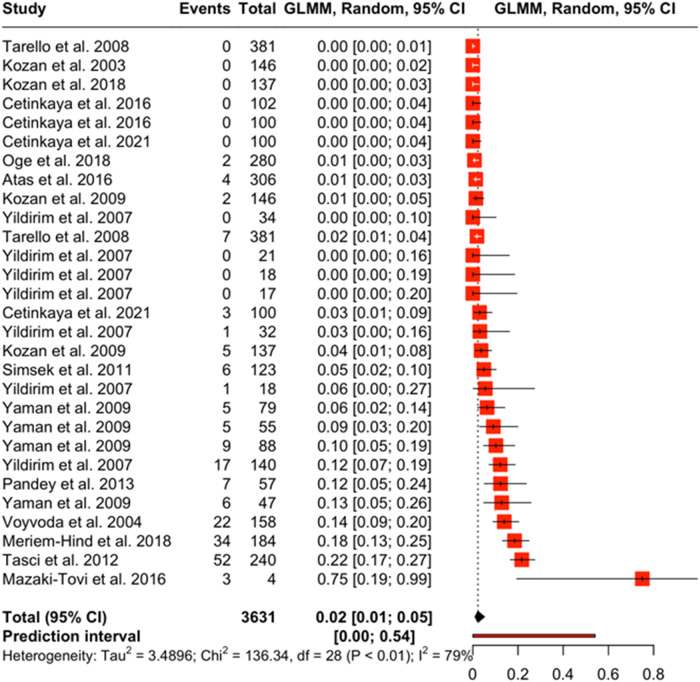
The forest plot shows the effect size of each observation against a predicted effect size (diamond symbol). This forest plot is from the microscopy diagnostic method moderator analysis.

Forest plots show the magnitude of difference between each observation in the moderator group. The boxes in the forest plot represent the point estimate of each observation and the horizontal lines represent the 95% confidence interval. The forest plot for PCR (Figure 4), estimated the prevalence of *Dirofilaria* spp. between 0% and 2% when this test type is used. The prediction interval for a new study using PCR is between 0% and 55%. Most of the observations that reported results from a PCR test have a point estimate close to zero; however, the observation from Mazaki-Tovi et al. (2016) shows a point estimate equal to 1 with a large confidence interval. This is because this observation had a 100% positivity rate, all 4 samples tested positive. The forest plot for rapid test (antigen) (Figure 5) estimated the prevalence of *Dirofilaria* spp. between 2% and 6% when this test type is used. The prediction interval for a new study using rapid test (antigen) is between 0% and 58%. The studies with the highest rate of positivity (Yaman et al. 2009; Elhamiani Khatat et al. 2017; Sari *et al.* 2013; Pasa *et al.* 2017) also have the highest point estimates. The forest plot for microscopy (Figure 6) estimated the prevalence of *Dirofilaria* spp. between 1% and 5% when this test type is used. The prediction interval for a new study using microscopy is between 0% and 54%.

### Publication bias and outlier analysis

**Figure 7.**
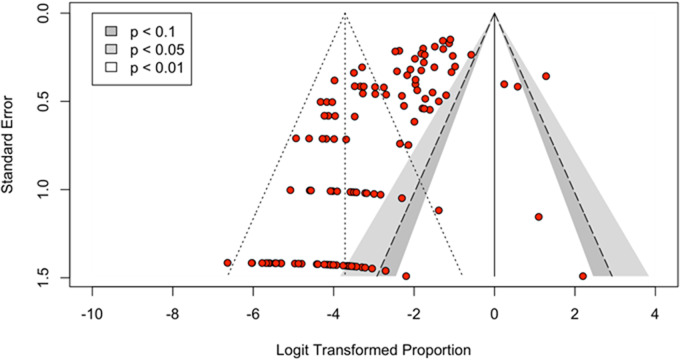
Funnel plot showing the relationship between the estimated effect size of each observation against the true effect size. When observations (red dots) are centred around 0 on the horizontal axis, the estimated effects are close to the true effect. This funnel, plot shows deviation in the estimated effect sizes from the true effect size.

The contour-enhanced funnel plot (Figure 7) is a scatter plot of the effect estimate, prevalence, against the standard error (Sterne et al., 2011) for the entire meta-analysis dataset. Most observations fall beyond the *p* < 0.1 range of the plot with a wide distribution of observations across the *x*-axis.

The trim-and-fill method is a statistical method that serves 2 purposes; first, it indicates the significance of the publication bias and it provides bias-adjusted results (Shi and Lin, 2019). In the first trim-and-fill analysis, performed on the ‘Meta-analysis dataset,’ the model added 53 studies, total observations, *k* = 185 and the estimated prevalence was 12.45% (95% CI: 9.12–16.78%; *I*^2^ = 87.1%, 95% CI of *I*^2^: 85.5–88.5%), *p* < 0.0001 suggests a likelihood of publication bias. The second trim-and-fill analysis was on the data set with the 39 previously identified outlier observations removed. This model added 36 studies, total observations, *k* = 131 and the estimated prevalence of this dataset was 12.33% (95% CI: 8.80–17.02%; *I*^2^ = 85.7%, 95% CI of *I*^2^: 83.5–87.6%), *p* < 0.0001.

## Discussion

There is a significant knowledge gap about *Dirofilaria* spp. in North Africa and the Middle East. This literature review and meta-analysis reviewed publications reporting cases of either *D. immitis* or *D. repens* in any host or vector species from 21 countries in Northern Africa and the Middle East. The data in the publications included in the model estimated a pooled prevalence of 2.4%. These findings show that there is transmission of *Dirofilaria* spp. in this geographic region, including to humans.

The meta-analysis model has a high degree of heterogeneity, *I*^2^ = 81.7%. Heterogeneity in meta-analysis is defined as variation in true effect size (prevalence) between publications (Higgins, 2008). Heterogeneity (*I*^2^) is calculated as a percentage of the variation across publications, which is due to differences in the findings of the publication and not due to chance (Higgins and Thompson, 2002; Thorlund et al., 2012). It is considered high when it is greater than 50% (Deeks et al., 2023). The heterogeneity in this meta-analysis is likely due to several factors including the small number of publications included in the analysis, and the small number of positive diagnoses for *D. immitis* or *D. repens* among included publications. The outlier analysis removed 39 observations and estimated the heterogeneity of the model equal to 0% as opposed to 81.7% when the outliers remained in the model. This provides strong evidence that the identified outliers are strong contributors to the heterogeneity of the dataset (Lin et al., 2017). The lower heterogeneity with outliers removed suggests that the studies being analysed are all estimating the same underlying population effect size, and any differences in effect sizes observed between studies are likely due to random sampling variability rather than true differences in the effects being studied. All observations identified as outliers did contain positive diagnostic events of *Dirofilaria* spp.; however, there was no pattern or trend based on country or diagnostic test type. More analysis is needed to understand how these observations influence the prevalence estimate.

The 2 trim-and-fill analysis investigated sources of the bias in the dataset by removing some of the extreme values and imputing new values to create a new estimate with less bias. The trimmed and filled datasets both estimated a prevalence of 12%, considerably higher than the 2.4 % estimated in the original model. Trim-and-fill attempts to fill in missing observations due to publication bias by estimating the values of those missing values (Shi and Lin, 2019). The fivefold increase in estimated prevalence from the trim-and-fill analysis suggests the true prevalence of *Dirofilaria* spp. might be much higher. This also supports the need for additional studies and increased surveillance. Additional analysis exploring sources of bias and risk of bias assessments would be beneficial.

Specifically, observations 93 and 33 indicated high influence and heterogeneity when visualized on the Baujat plot. The asymmetry in the funnel plot also indicates the presence of bias in this study. *Dirofilaria* spp. is reported in many countries and hosts, but model constraints dictated the inclusion criteria for this study and resulted in the exclusion of small studies. The final dataset is heavily weighted with studies from Türkiye. Funnel plot asymmetry can be a symptom of a number of characteristics in the dataset including publication bias, presence of studies with small sample size, chance, poor methodological design and true heterogeneity (Sterne et al., 2011).

Moderator analysis is an important component of meta-analysis because it allows exploration of drivers associated with the prevalence of *D. immitis* and *D. repens*. This analysis assessed 4 different moderators: continent of infection origin, host species, *Dirofilaria* spp. and diagnostic test method, and found a statistically significant difference in diagnostic test type as a moderator. Publications from Kuwait, Jordan, Israel, Iraq, Saudia Arabia and Tunisia occurred 1 time each in the dataset, less than the needed 3 occurrences of each variable value. The goal of this meta-analysis was to describe the prevalence of *Dirofilaria* spp. across the 21 countries of interest, so publications from these countries remained in the model to enhance geographic representation of publications. Inclusion of data from these countries might impact the heterogeneity and bias of the analysis. The decision to include publications from countries with fewer than 3 publications meant moderator analysis by country could not be performed; however, moderator analysis by continent was performed. This model did not detect a statistical difference in prevalence based on continent of infection origin, host species or *Dirofilaria* spp. in this dataset. However, there are important clinical and medical differences based on host and pathogen species. Differences in prevalence between continents are expected because of the difference in climate, but that was also not a statistically significant between group difference. The pooled prevalence in Asian countries was 2.5%, and 2.0% in African countries. Mosquito species competent for *Dirofilaria* spp. transmission are well documented in all of the countries included in this meta-analysis, but there are differences in humidity, population density, rainfall and pollution that might impact vector activity, how likely hosts are to come into contact with vectors and ultimately impact transmission.

With regard to host species, the meta-analysis dataset included more observations of sampled dogs (*n* = 127) than cats (*n* = 5). It is possible that a more balanced sample would produce different prevalence results. *D. immitis* and *D. repens* have different presentations in different hosts (Noack et al., 2021). *D. immitis* is of great importance in veterinary medicine and can be diagnosed using a point-of-care test. Diagnosis of *D. repens* often requires excision and identification of adult worms from nodules. The model did not detect a statistical difference in prevalence between host species included in the ‘Meta-analysis dataset’.

With regard to each species of *Dirofilaria* in the meta-analysis dataset, the model did not detect a statistical difference in the prevalence estimates of *D. immitis* or *D. repens. D. immitis* is often thought of as causing disease solely in dogs, but it causes clinical disease in cats and the effects can be devastating. *D. repens* is often thought of as a zoonotic disease, affecting both humans and animals. Preventatives for *D. repens* are available in some countries, but may not be available globally, especially in North Africa and the Middle East. Countries included in this analysis often experience ongoing conflict that jeopardizes stability and security, as well as health for both humans and animals. It is not surprising that countries with long-standing conflict like Libya, Syria and Yemen do not have publications on *Dirofilaria* spp. in the literature (Daw, 2021). Pathogens still circulate, and it is imperative that medical supplies and support reach areas in conflict to maintain the health of humans and animals.

Diagnostic test type was a key factor in estimating the prevalence of *Dirofilaria* spp. in this study – an expected finding due to inherent differences in test sensitivity, specificity and required operator skill. When multiple test methods were performed on the same animal, for example a rapid test and PCR, these were treated as independent tests because the results of one test did not impact the decision to provide a subsequent test. Differences in diagnostic tests are a key factor in understanding the findings of prevalence in the moderator analysis models, and their generalizability. The estimated prevalence using all diagnostic methods irrespective of the molecule/pathogen detected was 2.4%. The moderator analysis for the grouped diagnostic test types created models that account for each test’s sensitivity, specificity and the underlying sample size. PCR (0.7%) detected parasite DNA, and the lateral flow ELISAs (rapid test antigen) (3.9%) detected antigen of adult female worms. Microscopy morphologically identified microfilaria/adult worms (2.3%). The differences in prevalence between diagnostic testing method are not surprising. Rapid antigen tests using whole blood or serum from the animal is currently the gold standard for point-of-care diagnostics. PCR is a high sensitivity testing method but will only detect an infection in a blood sample if microfilaria are circulating in the blood stream. An animal recently infected with *D. immitis* or an infection of *D. repens* that is in a subcutaneous nodule are likely to not have circulating microfilaria that can be detected by PCR. However, PCR used when adult worms are the sample source can detect *Dirofilaria* spp. with a high degree of sensitivity. Microscopy has similar considerations to PCR, with added dependence on the skill of the person looking through the microscope. The sample type used in each of these test types is critically important and that is not something that was assessed with this analysis.

Commercially manufactured rapid tests are the most commonly used among publications in this study, but there is variation in availability of test kits by geographic locations, not all tests are licensed in all countries. This limits the ability to standardize testing across countries and locations. Most of the case reports in humans determined infection based on microscopic analysis of the physical worm or microfilaria taken from the patient, but they omitted reference to the key or standard that was used to identify the species in the sample. Some authors used multiple tests on the same sample of animals, for example a rapid test and PCR but did not indicate the degree of concordance between the two testing methods (Adanir et al., 2013; Omar et al., 2018). For this analysis, each test type was considered independently of any other test performed on the same sample of animals. There is a wide range of representation by each country included in the query. The literature search returned the most publications from Türkiye, but no publications from Mauritania, Libya or Sudan, for example.

This meta-analysis identified opportunities for improved reporting that would strengthen future meta-analytic approaches: (1) specify source/geographic location of definitive and intermediate host and vector samples, include coordinates if possible; (2) demographic details about where the host became infected if different from sampling location; (3) state what specimen was used in the diagnostic test (blood, serum, tissue); (4) the diagnostic tests used should include manufacturer name and full test name; (5) if performing microscopic or morphologic identification of *Dirofilaria* or mosquito vector species, include the citation or key that is used to determine specimen characteristics; (6) if multiple tests are performed on the same sample, indicate concordance/discordance.

The meta-analysis model did provide an estimate of *Dirofilaria* spp. prevalence for the 21 countries included in this study and also found variations in prevalence estimates. The overall prevalence was 2.4%, with outliers removed it was 1.0%, and with trim-and-fill analysis it was 12%. The drivers behind the variations in prevalence estimates warrant further investigation. Türkiye is heavily represented in the publications used in this analysis; a more balanced representation from countries in the region would provide an estimate that is more generalizable to the region. Additional studies with larger sample size are desirable to increase statistical power and representation across the region. The social and political landscape of North Africa and the Middle East might be a barrier to research and publication from some of these countries. The gaps and limitations identified in this study provide opportunities for future collaboration and research. This is the first estimate of *Dirofilaria* spp. for these countries; however, the true prevalence remains unknown. The model is limited by the data available in publications and inclusion criteria. This study highlighted gaps in research and opportunities for future research from this region on the topic of *Dirofilaria* spp. transmission. Foremost, adoption and adherence to recognized standards for testing, treatment and use of preventatives would enhance the welfare of dogs in this region by protecting them from canine heartworm disease. A standardized testing programme could also improve accuracy and timeliness of *Dirofilaria* spp. diagnosis because training and testing resources could be prepositioned within the country or jurisdiction.

## Supporting information

Izenour et al. supplementary material 1 Izenour et al. supplementary material

Izenour et al. supplementary material 2 Izenour et al. supplementary material

Izenour et al. supplementary material 3 Izenour et al. supplementary material

Izenour et al. supplementary material 4 Izenour et al. supplementary material

Izenour et al. supplementary material 5 Izenour et al. supplementary material

## Supplementary material

The supplementary material for this article can be found at https://doi.org/10.1017/S003118202500037X.
