# Supplementary material for: A meta-analysis on Dirofilaria immitis and Dirofilaria repens in countries of North Africa and the Middle East
Source: Parasitology. 2025 Apr 1;152(4):347–65. doi: 10.1017/S003118202500037X (PMC12186096; doi:10.1017/S003118202500037X)
Supplement: Izenour et al. supplementary material 1 — Izenour et al. supplementary material [file S003118202500037Xsup001.docx]

---

title: "Dirofilaria Meta-Analysis Middle East and North Africa -Subset1 Dataset"

author: "Katie Izenour"

date: "2024-08-18

output: word_document

---

```{r setup, include=FALSE}

knitr::opts_chunk$set(echo = TRUE)

```

```{r}

# Importing the dataset

#---- Libraries -----

library(readxl)

library(metasens)

library(metafor)

library(meta)

library(tidyverse)

library(rmarkdown)

if (!require("devtools")) {

install.packages("devtools")

}

devtools::install_github("MathiasHarrer/dmetar")

library(dmetar)

install_github("MathiasHarrer/dmetar", force=TRUE)

#---- Import Statement -----

subset1 <- read_excel("C:/Users/XXXXX/MetaAnalysisModelRecords.xlsx", sheet='subset1')

# ---- Data Cleaning ----

# Takes all character variables and makes factors

subset1[sapply(subset1, is.character)] = lapply(subset1[sapply(subset1, is.character)], as.factor)

str(subset1) #Checking the structure

```

```{r meta}

m.propsub <- metaprop(event = subset1$npositive,

n = subset1$totalsample,

studlab = as.character(subset1$recordID),

prediction = TRUE,

data = subset1,

method = "GLMM",

sm = "PLOGIT",

fixed = FALSE,

random = TRUE,

hakn = TRUE,

title = "Meta-Results - Data= Subset1")

summary(m.propsub)

```

```{r outliers}

m.propsub2=find.outliers(m.propsub)

m.propsub2

```

```{r influence}

propsub.inf = InfluenceAnalysis(m.propsub, random = TRUE)

png(filename = "C:\\Users\\XXXXXX\\Sub_Baujat.png", width=5000,height=8000,res=400)

plot(propsub.inf, "baujat") # Can change different plots as necessary

dev.off() #stops from saving more into the PNG file

```

```{r moderators}

#---- Moderator Analysis ----

# All variables are represented

# Some of the variables may not be suitable for final inclusion.

#1 - Diagnostic Method, all values for diagnostic methods

update(m.propsub,

subgroup = DxMethod,

tau.common = FALSE)

#2 - Substrate, the biologic agent being tested/detected

update(m.propsub,

subgroup = Substrate,

tau.common = FALSE)

#3 - DxMethodGroup, aggregation of DXMethod variable

update(m.propsub,

subgroup = DxMethodGroup,

tau.common = FALSE)

#4 - TestCombo, indication if a single test was used on the sample, or if multiple tests were used on the same sample.

update(m.propsub,

subgroup = TestCombo,

tau.common = FALSE)

#5 - DxSample, sample from the animal used in the diagnostic test

update(m.propsub,

subgroup = DxSample,

tau.common = FALSE)

#6 - DXSampleGroup, aggregation of DX sample variable.

update(m.propsub,

subgroup = DxSampleGroup,

tau.common = FALSE)

#7 - Organ_structure_involved indicates location of worms/sample, or if sample was collected for surveillance

update(m.propsub,

subgroup = organ_structure_involved,

tau.common = FALSE)

#8 - structureGroup, aggregation of organ_structure_involved'

update(m.propsub,

subgroup = structureGroup,

tau.common = FALSE)

#9 - StructureGroup2, aggregation of structureGroup

update(m.propsub,

subgroup = structureGroup2,

tau.common = FALSE)

#10 - HostSpecies, host the sample was collected from

update(m.propsub,

subgroup = HostSpecies,

tau.common = FALSE)

#11 - HostGroup, aggregation of HostSpecies Variable

update(m.propsub,

subgroup = HostGroup,

tau.common = FALSE)

#12 - HostGroup2, aggregation of HostGroup

update(m.propsub,

subgroup = HostGroup2,

tau.common = FALSE)

#13 - DirofilariaSpecies, species detected by test

update(m.propsub,

subgroup = DirofilariaSpecies,

tau.common = FALSE)

#14 - Country data

update(m.propsub,

subgroup = Country,

tau.common = FALSE)

#15 - City data

#update(m.propsub,

# subgroup = City,

# tau.common = FALSE)

#16 - continent data

update(m.propsub,

subgroup = Continent,

tau.common = FALSE)

```

```{r Subgroup Forest}

#Forest Plot of diagnostic group moderator analysis

#Step 1 - make individual datasets of the subgroups for each level of diagnostic group

DxGroupPCR <- subset1[subset1$DxMethodGroup == "PCR", ]

DxGroupRapid <- subset1[subset1$DxMethodGroup == "Rapid Test (antigen)", ]

DxGroupMicro <- subset1[subset1$DxMethodGroup == "Microscopy", ]

#Step 2 - Make individual Random effects models for each new subgroup dataset

#then output to .png file

m.PCRsub <- metaprop(event = DxGroupPCR$npositive,

n = DxGroupPCR$totalsample,

studlab = as.character(DxGroupPCR$recordID),

prediction = TRUE,

data = DxGroupPCR,

method = "GLMM",

sm = "PLOGIT",

fixed = FALSE,

random = TRUE,

hakn = TRUE,

title = "Meta-Results - Data= DxGroupPCR")

summary(m.PCRsub)

png(filename = "C:\\XXXXXXX\\Forest_PCRDxGroup.png", width=1200,height=1400,res=150)

forest(m.PCRsub)

dev.off()

####################################################################

m.Rapidsub <- metaprop(event = DxGroupRapid$npositive,

n = DxGroupRapid$totalsample,

studlab = as.character(DxGroupRapid$recordID),

prediction = TRUE,

data = DxGroupRapid,

method = "GLMM",

sm = "PLOGIT",

fixed = FALSE,

random = TRUE,

hakn = TRUE,

title = "Meta-Results - Data= DxGroupRapid")

summary(m.Rapidsub)

png(filename = "C:\\XXXXX\\Forest_RapidDxGroup.png", width=1030,height=2000,res=120)

forest(m.Rapidsub)

dev.off()

#####################################################################

m.Microsub <- metaprop(event = DxGroupMicro$npositive,

n = DxGroupMicro$totalsample,

studlab = as.character(DxGroupMicro$recordID),

prediction = TRUE,

data = DxGroupMicro,

method = "GLMM",

sm = "PLOGIT",

fixed = FALSE,

random = TRUE,

hakn = TRUE,

title = "Meta-Results - Data= DxGroupMicro")

summary(m.Microsub)

png(filename = "C:\\XXXXXX\\Forest_MicroDxGroup.png", width=1200,height=1080,res=140)

forest(m.Microsub)

dev.off()

```

```{r publication bias}

#---- Contoured Funnel Plot ----

# Define fill colors for contour

col.contour = c("gray75", "gray85", "gray95")

# Generate funnel plot

funnel(m.propsub,

xlim = c(-10,4),

contour = c(0.9, 0.95, 0.99),

col.contour = col.contour,

studlab = T)

# Add a legend

legend(x = -10, y = 0,

legend = c("p < 0.1", "p < 0.05", "p < 0.01"),

fill = col.contour)

# Add a title

title("Contour-Enhanced Funnel Plot, Subset1 Data")

# ---- Peter's Test of Funnel Plot ----

metabias(m.propsub, method.bias = "peters")

# ---- Trim & Fill ----

m.propsub$I2

# Two trim and fill analysis were conducted in light of the high heterogeneity - one on entire data set, one with identified outliers removed

trim.prop = trimfill(m.propsub)

trim.prop2 = trimfill(update(m.propsub,

subset = -c(2,8,10,33,35,45,46,48,49,51,58,59,60,64,65,66,67,68,69,78, 80,81,83,93,94,96,97,98,99,100,101,102,103,105,112,124,129,130)))

summary(trim.prop) # 53 studies added

summary(trim.prop2) # 35 Additional studies added

```
