# Supplementary material for: A meta-analysis on Dirofilaria immitis and Dirofilaria repens in countries of North Africa and the Middle East
Source: Parasitology. 2025 Apr 1;152(4):347–65. doi: 10.1017/S003118202500037X (PMC12186096; doi:10.1017/S003118202500037X)
Supplement: Izenour et al. supplementary material 2 — Izenour et al. supplementary material [file S003118202500037Xsup002.docx]

**S1. Publication query search parameters**PubMed Search Query

(("D. immitis" or "Dirofilaria immitis"or "D. repens"or "Dirofilaria repens"or"mosquito")AND("Middle East" or "North Africa" or "Mediterranean" or "Morocco" or "Tunisia" or "Algeria" or "Egypt" or "Libya" or "Mauritania" or "Sudan" or "Oman" or "Saudi Arabia" or "Yemen" or "Iraq" or "Jordan" or "Syria" or "Lebanon" or "Israel" or “Palestine” or “Occupied Palestinian Territory” or “Kuwait” or "Qatar" or "United Arab Emirates" or "United Arab Emirate" or "Bahrain" or “Türkiye” or "Turkey"))

Web of Science Search Query

(((Middle East) OR (North Africa)OR(Morocco)OR(Tunisia)OR(Algeria)OR(Egypt)OR(Libya)OR(Mauritania)OR(Sudan)OR(Oman)OR(Saudi Arabia)OR(Yemen)OR(Iraq)OR(Jordan)OR(Syria)OR(Lebanon)OR(Israel)OR(Kuwait)OR(Qatar)OR(United Arab Emirates)OR(United Arab Emirate) OR(Palestine)OR(Occupied Palestinian Territory)OR(Bahrain)OR(Turkey)OR(Türkiye)OR(Mediterranean)) and ((heartworm) OR (dirofilaria immitis) OR (d immitis) OR (dirofilaria repens) OR (mosquito) OR (d repens)))
