# Supplementary material for: A meta-analysis on Dirofilaria immitis and Dirofilaria repens in countries of North Africa and the Middle East
Source: Parasitology. 2025 Apr 1;152(4):347–65. doi: 10.1017/S003118202500037X (PMC12186096; doi:10.1017/S003118202500037X)
Supplement: Izenour et al. supplementary material 3 — Izenour et al. supplementary material [file S003118202500037Xsup003.docx]

**Supplement 2**

**All publications reporting detection of *D. immitis* or *D. repens* by country with detection technique, ‘full dataset’**

| **Publication (first author year)** | **Country** | **City/Province/Region** | **Species** | **Host** | **Sample Source** | **Total Sample** | **Knotts/ Modified Knotts** | | **Native Technique** | | **Difil** | | **Microscopy Smear** | | **Whole Worms/morphology** | | **non- specific** | | **Membrane Filtration-Acid Phosphate Histochemical Staining** | | **Antigen i.e. Rapid ELISA Antigen Tests (SNAP**^®^ **Tests)** | | **ELISA (Green Spring D. immitis IgG)** | | **ELISA non-specific** | | **PCR** | | **Visual Inspection not otherwise specified** | |
| --- | --- | --- | --- | --- | --- | --- | --- | --- | --- | --- | --- | --- | --- | --- | --- | --- | --- | --- | --- | --- | --- | --- | --- | --- | --- | --- | --- | --- | --- | --- |
| **(Abdel-Rahman et al. 2008)** | Egypt | Assuit Governorate | *D. repens* | human | worm and blood | 1 |  | |  | |  | | 0 | | 1 | |  | |  | |  | |  | |  | | 1 | |  | |
|  |  | Assuit Governorate | *D. repens* | human | worm and blood | 2 |  | |  | |  | | 0 | | 2 | |  | |  | |  | |  | |  | |  | |  | |
| **(Abdullah et al. 2021)** | Egypt | Cairo | *D. repens* | dog | blood | 203 |  | |  | |  | |  | |  | |  | |  | |  | |  | |  | | 2 | |  | |
| **(Abo-Aziza et al. 2022)** | Egypt | Cairo | *D. repens* | horse | blood | 311 |  | |  | |  | |  | |  | |  | |  | |  | |  | |  | |  | |  | |
|  |  |  |  |  |  |  |  |  |  |  |  |  |  |  |  |  |  |  |  |  |  |  |  |  |  |  |  |  |  |  |
|  |  | Beni-Suef | *D. repens* | horse | blood | 9 |  | |  | |  | |  | |  | |  | |  | |  | |  | |  | |  | |  | |
|  |  |  |  |  |  |  |  |  |  |  |  |  |  |  |  |  |  |  |  |  |  |  |  |  |  |  |  |  |  |  |
|  |  | Beni-Suef | *D. repens* | donkey | blood, worms | 22 |  | |  | |  | |  | |  | |  | |  | |  | |  | |  | | 1* | |  | |
|  |  |  |  |  |  |  |  |  |  |  |  |  |  |  |  |  |  |  |  |  |  |  |  |  |  |  |  |  |  |  |
|  |  | Al-Faiyum | *D. repens* | donkey | blood, worms | 87 |  | |  | |  | |  | |  | |  | |  | |  | |  | |  | |  | |  | |
| **(Adanir et al. 2013)** | Türkiye | Burdur | *D. immitis* | dog | blood | 142 |  | |  | |  | |  | |  | |  | |  | | 31 | |  | |  | |  | |  | |
|  |  | Burdur | *D. immitis* | dog | blood | 31 | 20 | | 17 | |  | |  | |  | |  | |  | |  | |  | |  | |  | |  | |
| **(Al-Kappany et al. 2011)** | Egypt | Abou-rawah | *D. immitis* | cat | blood | 174 |  | |  | |  | |  | |  | |  | |  | | 6 | |  | |  | |  | |  | |
| **(Atas et al. 2018)** | Türkiye | Sivas | *D. immitis* | dog | blood | 306 | 4 | |  | |  | |  | |  | |  | |  | | 9 | |  | |  | | 9 | |  | |
| **(Aykur et al. 2021)** | Türkiye | Izmir | *D. immitis* | human | worms | 1 |  | |  | |  | |  | | 1 | |  | |  | |  | |  | |  | | 1 | |  | |
| **(Biskin et al. 2010)** | Türkiye | Kayseri | *D. immitis* | *Culex pipiens* | mosquito pools | 205 mosquitoes |  | |  | |  | |  | |  | |  | |  | |  | |  | |  | | 0 of 34 head-thorax pools positive | |  | |
|  |  |  |  |  |  |  |  |  |  |  |  |  |  |  |  |  |  |  |  |  |  |  |  |  |  |  | 0 of 34 abdomen pools positive | |  |  |
|  |  |  |  | *Aedes vexans* | mosquito pools | 96 mosquitoes |  | |  | |  | |  | |  | |  | |  | |  | |  | |  | | 1 of 54 head-thorax pools positive | |  | |
|  |  |  |  |  |  |  |  |  |  |  |  |  |  |  |  |  |  |  |  |  |  |  |  |  |  |  | 1 of 54 abdomen pools positive | |  |  |
| **(Baneth et al. 2002)** | Israel | Nahariya | *D. repens* | dog | blood | 1 | 1 | |  | |  | | 1 | |  | |  | |  | | 0 | |  | |  | | positive *D. repens* primers, negative *D. immitis* primers | |  | |
| **(Beden et al. 2007)** | Türkiye | None specified | *D. repens* | human | worms | 1 |  | |  | |  | |  | | 1 | |  | |  | |  | |  | |  | |  | |  | |
| **(Ceribasi and Simsek 2012)** | Türkiye | Bursa Province | *D. immitis* | dog | worms, body organs, blood | 1 |  | |  | |  | |  | |  | | 1 | |  | |  | |  | |  | | 1 | | 1 | |
| **(Cetinkaya et al. 2016)** | Türkiye | Istanbul | *D. immitis* | dog | blood | 100 |  | |  | |  | | 0 | |  | |  | |  | | 0 | |  | |  | | 0 | |  | |
|  |  | Edirne | *D. immitis* | dog | blood | 102 |  | |  | |  | | 0 | |  | |  | |  | | 15 | |  | |  | | 5 | |  | |
|  |  | Tekirdag | *D. immitis* | dog | blood | 100 |  | |  | |  | | 0 | |  | |  | |  | | 1 | |  | |  | | 0 | |  | |
|  |  | Kirklareli | *D. immitis* | dog | blood | 100 |  | |  | |  | | 3 | |  | |  | |  | | 11 | |  | |  | | 6 | |  | |
| **(Ceylan et al. 2021)** | Türkiye | Adana | *D. immitis* | dog | blood | 31 |  | |  | |  | |  | |  | |  | |  | | 0 | |  | |  | |  | |  | |
|  |  | Ankara |  |  |  | 7 |  | |  | |  | |  | |  | |  | |  | | 0 | |  | |  | |  | |  | |
|  |  | Antalya |  |  |  | 13 |  | |  | |  | |  | |  | |  | |  | | 0 | |  | |  | |  | |  | |
|  |  | Aydin |  |  |  | 26 |  | |  | |  | |  | |  | |  | |  | | 1 | |  | |  | |  | |  | |
|  |  | Balikesir |  |  |  | 11 |  | |  | |  | |  | |  | |  | |  | | 1 | |  | |  | |  | |  | |
|  |  | Bursa |  |  |  | 7 |  | |  | |  | |  | |  | |  | |  | | 0 | |  | |  | |  | |  | |
|  |  | Denizli |  |  |  | 10 |  | |  | |  | |  | |  | |  | |  | | 0 | |  | |  | |  | |  | |
|  |  | Istanbul |  |  |  | 25 |  | |  | |  | |  | |  | |  | |  | | 0 | |  | |  | |  | |  | |
|  |  | Izmir |  |  |  | 12 |  | |  | |  | |  | |  | |  | |  | | 0 | |  | |  | |  | |  | |
|  |  | Kocaeli |  |  |  | 36 |  | |  | |  | |  | |  | |  | |  | | 1 | |  | |  | |  | |  | |
|  |  | Mugla |  |  |  | 29 |  | |  | |  | |  | |  | |  | |  | | 0 | |  | |  | |  | |  | |
|  |  | Sakarya |  |  |  | 41 |  | |  | |  | |  | |  | |  | |  | | 0 | |  | |  | |  | |  | |
| **(Chabchoub et al. 2003)** | Tunisia | Tunis | *D. immitis* | dog | Not specified | 127 |  | |  | |  | |  | |  | |  | |  | |  | |  | | 6 | |  | |  | |
| **(Chazan et al. 2001)** | Israel | None specified | *D. repens* | human | worms | 1 |  | |  | |  | |  | | 1 | |  | |  | |  | |  | |  | |  | |  | |
| **(Chopra et al. 2004)** | Saudi Arabia | Al-Hassa | *D. repens* | human | worms | 1 |  | |  | |  | |  | | 1 | |  | |  | |  | |  | |  | |  | |  | |
| **(Colak et al. 2020)** | Türkiye | None specified | *D. immitis* | dog | urine | 1 |  | |  | |  | | 1 | |  | |  | |  | |  | |  | | 1 | |  | |  | |
| **(Dababo et al. 2022)** | Saudi Arabia | None specified | *D. repens* | human | worms | 1 |  | |  | |  | |  | | 1 | |  | |  | |  | |  | |  | |  | |  | |
| **(Demirci et al. 2021)** | Türkiye | Aras Valley | *D. immitis* | *Aedes caspius* |  | 806 |  | |  | |  | |  | |  | |  | |  | |  | |  | |  | | 187 | |  | |
|  |  | Aras Valley | *D. immitis* | *Aedes vexans* |  | 16 |  | |  | |  | |  | |  | |  | |  | |  | |  | |  | | 2 | |  | |
|  |  | Aras Valley | *D. immitis* | *Anopheles hyrcanus* |  | 16 |  | |  | |  | |  | |  | |  | |  | |  | |  | |  | | 1 | |  | |
|  |  | Aras Valley | *D. immitis* | *Anopheles maculipennis s.l* |  | 36 |  | |  | |  | |  | |  | |  | |  | |  | |  | |  | | 8 | |  | |
|  |  | Aras Valley | *D. immitis* | *Culex pipiens* |  | 24 |  | |  | |  | |  | |  | |  | |  | |  | |  | |  | | 2 | |  | |
|  |  | Aras Valley | *D. immitis* | *Culex theileri* |  | 128 |  | |  | |  | |  | |  | |  | |  | |  | |  | |  | | 38 | |  | |
|  |  | Aras Valley | *D. immitis* | *Culiseta annulata* |  | 8 |  | |  | |  | |  | |  | |  | |  | |  | |  | |  | | 0 | |  | |
|  |  | Aras Valley | *D. repens* | *Aedes caspius* |  | 806 |  | |  | |  | |  | |  | |  | |  | |  | |  | |  | | 169 | |  | |
|  |  | Aras Valley | *D. repens* | *Aedes vexans* |  | 16 |  | |  | |  | |  | |  | |  | |  | |  | |  | |  | | 3 | |  | |
|  |  | Aras Valley | *D. repens* | *Anopheles hyrcanus* |  | 16 |  | |  | |  | |  | |  | |  | |  | |  | |  | |  | | 3 | |  | |
|  |  | Aras Valley | *D. repens* | *Anopheles maculipennis s.l* |  | 36 |  | |  | |  | |  | |  | |  | |  | |  | |  | |  | | 4 | |  | |
|  |  | Aras Valley | *D. repens* | *Culex pipiens* |  | 24 |  | |  | |  | |  | |  | |  | |  | |  | |  | |  | | 4 | |  | |
|  |  | Aras Valley | *D. repens* | *Culex theileri* |  | 128 |  | |  | |  | |  | |  | |  | |  | |  | |  | |  | | 34 | |  | |
|  |  | Aras Valley | *D. repens* | *Culiseta annulata* |  | 8 |  | |  | |  | |  | |  | |  | |  | |  | |  | |  | | 0 | |  | |
|  |  | Aras Valley | *D. immitis and D. repens* | *Aedes caspius* |  | 806 |  | |  | |  | |  | |  | |  | |  | |  | |  | |  | | 114 | |  | |
|  |  | Aras Valley | *D. immitis and D. repens* | *Aedes vexans* |  | 16 |  | |  | |  | |  | |  | |  | |  | |  | |  | |  | | 1 | |  | |
|  |  | Aras Valley | *D. immitis and D. repens* | *Anopheles hyrcanus* |  | 16 |  | |  | |  | |  | |  | |  | |  | |  | |  | |  | | 1 | |  | |
|  |  | Aras Valley | *D. immitis and D. repens* | *Anopheles maculipennis s.l* |  | 36 |  | |  | |  | |  | |  | |  | |  | |  | |  | |  | | 6 | |  | |
|  |  | Aras Valley | *D. immitis and D. repens* | *Culex pipiens* |  | 24 |  | |  | |  | |  | |  | |  | |  | |  | |  | |  | | 0 | |  | |
|  |  | Aras Valley | *D. immitis and D. repens* | *Culex theileri* |  | 128 |  | |  | |  | |  | |  | |  | |  | |  | |  | |  | | 23 | |  | |
|  |  | Aras Valley | *D. immitis and D. repens* | *Culiseta annulata* |  | 8 |  | |  | |  | |  | |  | |  | |  | |  | |  | |  | | 0 | |  | |
| **(Dyab et al. 2015)** | Egypt | El Nikhila | *D. immitis* | *Culex* sp. |  | 10 pools |  | |  | |  | |  | |  | |  | |  | |  | |  | |  | | 2 pools | |  | |
|  |  | El Nikhila | *D. immitis* | *Aedes* sp. |  | 5 pools |  | |  | |  | |  | |  | |  | |  | |  | |  | |  | | 0 pools | |  | |
|  |  | El Nikhila | *D. immitis* | *Anopheles* sp. |  | 5 pools |  | |  | |  | |  | |  | |  | |  | |  | |  | |  | | 0 pools | |  | |
|  |  | El Nikhila | *D. repens* | *Culex* sp. |  | 10 pools |  | |  | |  | |  | |  | |  | |  | |  | |  | |  | | 1 pool | |  | |
|  |  | El Nikhila | *D. repens* | *Anopheles* sp. |  | 5 pools |  | |  | |  | |  | |  | |  | |  | |  | |  | |  | | 0 pools | |  | |
|  |  | El Nikhila | *D. repens* | *Aedes* sp. |  | 5 pools |  | |  | |  | |  | |  | |  | |  | |  | |  | |  | | 1 pool | |  | |
|  |  | El Matiaa | *D. immitis* | *Culex* sp. |  | 10 pools |  | |  | |  | |  | |  | |  | |  | |  | |  | |  | | 0 pools | |  | |
|  |  | El Matiaa | *D. immitis* | *Aedes* sp. |  | 5 pools |  | |  | |  | |  | |  | |  | |  | |  | |  | |  | | 0 pools | |  | |
|  |  | El Matiaa | *D. immitis* | *Anopheles* sp. |  | 5 pools |  | |  | |  | |  | |  | |  | |  | |  | |  | |  | | 0 pools | |  | |
|  |  | El Matiaa | *D. repens* | *Culex* sp. |  | 10 pools |  | |  | |  | |  | |  | |  | |  | |  | |  | |  | | 0 pools | |  | |
|  |  | El Matiaa | *D. repens* | *Anopheles* sp. |  | 5 pools |  | |  | |  | |  | |  | |  | |  | |  | |  | |  | | 1 pool | |  | |
|  |  | El Matiaa | *D. repens* | *Aedes* sp. |  | 5 pools |  | |  | |  | |  | |  | |  | |  | |  | |  | |  | | 0 pools | |  | |
|  |  | Sahel | *D. immitis* | *Culex* sp. |  | 10 pools |  | |  | |  | |  | |  | |  | |  | |  | |  | |  | | 1 pool | |  | |
|  |  | Sahel | *D. repens* | *Culex* sp. |  | 10 pools |  | |  | |  | |  | |  | |  | |  | |  | |  | |  | | 0 pools | |  | |
|  |  | Seleem | *D. immitis* | *Anopheles* sp. |  | 5 pools |  | |  | |  | |  | |  | |  | |  | |  | |  | |  | | 0 pools | |  | |
|  |  | Seleem | *D. immitis* | *Aedes* sp. |  | 5 pools |  | |  | |  | |  | |  | |  | |  | |  | |  | |  | | 0 pools | |  | |
|  |  | Seleem | *D. repens* | *Anopheles* sp. |  | 5 pools |  | |  | |  | |  | |  | |  | |  | |  | |  | |  | | 0 pools | |  | |
|  |  | Seleem | *D. repens* | *Aedes* sp. |  | 5 pools |  | |  | |  | |  | |  | |  | |  | |  | |  | |  | | 0 pools | |  | |
| **(Elhamiani Khatat et al. 2017)** | Morocco | Tangier | *D. immitis* | dog | blood | 4 |  | |  | |  | |  | |  | |  | |  | | 0 | |  | |  | |  | |  | |
|  |  | Oujda | *D. immitis* | dog | blood | 5 |  | |  | |  | |  | |  | |  | |  | | 1 | |  | |  | |  | |  | |
|  |  | Sidi Kacem | *D. immitis* | dog | blood | 78 |  | |  | |  | |  | |  | |  | |  | | 28 | |  | |  | |  | |  | |
|  |  | Rabat | *D. immitis* | dog | blood | 57 |  | |  | |  | |  | |  | |  | |  | | 1 | |  | |  | |  | |  | |
|  |  | Benslimane | *D. immitis* | dog | blood | 25 |  | |  | |  | |  | |  | |  | |  | | 5 | |  | |  | |  | |  | |
|  |  | Marrakech | *D. immitis* | dog | blood | 32 |  | |  | |  | |  | |  | |  | |  | | 0 | |  | |  | |  | |  | |
|  |  | Sahara | *D. immitis* | dog | blood | 16 |  | |  | |  | |  | |  | |  | |  | | 0 | |  | |  | |  | |  | |
| **(Erkilic et al. 2019)** | Türkiye | Kars | unknown (Dirofilariasis) | cat | serum | 78 |  | |  | |  | |  | |  | |  | |  | |  | |  | | 23 | |  | |  | |
|  |  | Sarikamis | unknown (Dirofilariasis) | cat | serum | 30 |  | |  | |  | |  | |  | |  | |  | |  | |  | | 5 | |  | |  | |
|  |  | Arpacay | unknown (Dirofilariasis) | cat | serum | 24 |  | |  | |  | |  | |  | |  | |  | |  | |  | | 2 | |  | |  | |
|  |  | Selim | unknown (Dirofilariasis) | cat | serum | 18 |  | |  | |  | |  | |  | |  | |  | |  | |  | | 1 | |  | |  | |
| **(Fleck et al. 2009)** | Tunisia | None specified | *D. repens* | human | worms | 1 |  | |  | |  | |  | | 1 | |  | |  | |  | |  | |  | |  | |  | |
| **(Govrin-Yehudain et al. 2017)** | Israel | None specified | *D. repens* | human | worms | 1 |  | |  | |  | |  | | 1 | |  | |  | |  | |  | |  | |  | |  | |
| **(Gutierrez et al. 1995)** | Israel | Acre | *D. repens* | human | worms | 1 |  | |  | |  | |  | | 1 | |  | |  | |  | |  | |  | |  | |  | |
| **(Guven et al. 2017)** | Türkiye | Erzurum | *D. immitis* | dog | blood | 133 |  | |  | |  | |  | |  | |  | |  | |  | |  | |  | | 2 | |  | |
|  |  | Erzurum | *D. repens* | dog | blood | 133 |  | |  | |  | |  | |  | |  | |  | |  | |  | |  | | 0 | |  | |
| **(Harrus et al. 1999)** | Israel | None specified | *D. repens* | dog | blood and lymphnode | 1 |  | |  | |  | | 1 | |  | |  | |  | | 0 | |  | |  | | 1 | |  | |
| **(Hira et al. 2008)** | Kuwait | Kuwait City | *D. repens* | human | worms | 1 |  | |  | |  | |  | | 1 | |  | |  | |  | |  | |  | |  | |  | |
| **(Hira et al. 1994)** | Kuwait | None specified | *D. repens* | human | worms | 1 |  | |  | |  | |  | | 1 | |  | |  | |  | |  | |  | |  | |  | |
| **(Icen et al. 2011)** | Türkiye | Diyarbakir | *D. immitis* | dog | blood | 82 |  | |  | |  | |  | |  | |  | |  | | 2 | |  | |  | |  | |  | |
| **(Kaouech et al. 2010)** | Tunisia | None specified | *D. repens* | human | worms | 1 |  | |  | |  | |  | | 1 | |  | |  | |  | |  | |  | |  | |  | |
| **(Izenour et al. 2022)** | Egypt |  | *D. immitis* | dog | blood | 114 |  | |  | |  | |  | |  | |  | |  | | 0 | |  | |  | | 0 | |  | |
|  |  |  | *D. repens* | dog | blood | 114 |  | |  | |  | |  | |  | |  | |  | |  | |  | |  | | 0 | |  | |
| **(Koltas et al. 2002)** | Türkiye | Adana | *D. repens* | human | worms | 1 |  | |  | |  | |  | | 1 | |  | |  | |  | |  | |  | |  | |  | |
| **(Köse and Erdogan 2012)** | Türkiye | Ankara | *D. immitis* | dog | blood | 19 |  | |  | |  | |  | |  | |  | |  | | 0 | |  | |  | |  | |  | |
|  |  | Eskisehir | *D. immitis* | dog | blood | 33 |  | |  | |  | |  | |  | |  | |  | | 5 | |  | |  | |  | |  | |
|  |  | Afyonkara-hisar | *D. immitis* | dog | blood | 34 |  | |  | |  | |  | |  | |  | |  | | 6 | |  | |  | |  | |  | |
|  |  | Konya | *D. immitis* | dog | blood | 51 |  | |  | |  | |  | |  | |  | |  | | 1 | |  | |  | |  | |  | |
|  |  | Nevsehir | *D. immitis* | dog | blood | 28 |  | |  | |  | |  | |  | |  | |  | | 4 | |  | |  | |  | |  | |
|  |  | Aksaray | *D. immitis* | dog | blood | 25 |  | |  | |  | |  | |  | |  | |  | | 3 | |  | |  | |  | |  | |
|  |  | Nigde | *D. immitis* | dog | blood | 25 |  | |  | |  | |  | |  | |  | |  | | 1 | |  | |  | |  | |  | |
|  |  | Kars | *D. immitis* | dog | blood | 42 |  | |  | |  | |  | |  | |  | |  | | 4 | |  | |  | |  | |  | |
|  |  | Malatya | *D. immitis* | dog | blood | 23 |  | |  | |  | |  | |  | |  | |  | | 2 | |  | |  | |  | |  | |
|  |  | Mersin | *D. immitis* | dog | blood | 37 |  | |  | |  | |  | |  | |  | |  | | 1 | |  | |  | |  | |  | |
| **(Kozan et al. 2007)** | Türkiye | Afyonkarahisar | *Dirofilaria sp.* | dog | blood | 137 | 5 | |  | |  | |  | |  | |  | |  | |  | |  | |  | |  | |  | |
|  |  | Eskisehir | *Dirofilaria sp.* | dog | blood | 146 | 2 | |  | |  | |  | |  | |  | |  | |  | |  | |  | |  | |  | |
| **(Kutluturk et al. 2016)** | Türkiye | Marmara | *D. repens* | human | worms | 3 |  | |  | |  | |  | |  | |  | |  | |  | |  | |  | | 2 | |  | |
| **(Latifoglu et al. 2002)** | Türkiye | None specified | *D. repens* | human | worms | 1 |  | |  | |  | |  | | 1 | |  | |  | |  | |  | |  | |  | |  | |
| **(Makni et al. 2007)** | Tunisia | Sfax | *D. repens* | human | worms | 1 |  | |  | |  | |  | | 1 | |  | |  | |  | |  | |  | |  | |  | |
| **(Mazaki-Tovi et al. 2016)** | Israel | None specified | *D. repens* | dog | blood | 4 |  | |  | |  | | 3 | |  | |  | |  | |  | |  | |  | | 4 | |  | |
| **(Meriem-Hind and Mohamed 2009)** | Algeria | Algiers | *D. immitis* | dog | blood | 184 | 34 | |  | |  | |  | |  | |  | |  | | 45 | |  | |  | |  | |  | |
| **(Mittal et al. 2008)** | United Arab Emirates | Dubai | *D. repens* | human | worms | 1 |  | |  | |  | |  | |  | | 1 | |  | |  | |  | |  | |  | |  | |
| **(Mrad et al. 1999)** | Tunisia | None specified | *D. repens* | human | worms | 1 |  | |  | |  | |  | |  | | 1 | |  | |  | |  | |  | |  | |  | |
| **(Munichor et al. 2001)** | Israel | None specified | *D. repens* | human | worms | 1 |  | |  | |  | |  | | 1 | |  | |  | |  | |  | |  | |  | |  | |
| **(Obaidat and Alshehabat 2018)** | Jordan | None specified | *D. immitis* | dog | serum | 161 |  | |  | |  | |  | |  | |  | |  | | 0 | |  | |  | |  | |  | |
| **(Oge et al. 2003)** | Türkiye | Ankara | *D. immitis* | dog | blood | 280 |  | |  | |  | |  | |  | |  | | 2 | | 24 | |  | |  | |  | |  | |
| **(Omar et al. 2018)** | Saudi Arabia | Riyadh | *D. immitis* | dog | blood | 294 |  | |  | |  | |  | |  | |  | |  | | 23 | | 40 | |  | |  | |  | |
|  |  |  |  | cat | blood | 190 |  | |  | |  | |  | |  | |  | |  | | 3 | | 4 | |  | |  | |  | |
| **(Oncel and Vural 2005)** | Türkiye | Izmir | *D. immitis* | dog | blood | 117 |  | |  | |  | |  | |  | |  | |  | | 0 | |  | |  | |  | |  | |
|  |  | Istanbul | *D. immitis* | dog | blood | 263 |  | |  | |  | |  | |  | |  | |  | | 4 | |  | |  | |  | |  | |
| **(Otranto et al. 2019)** | Iraq | None specified | *D. immitis* | jackal | blood | 55 |  | |  | |  | |  | |  | |  | |  | |  | |  | |  | | 2 | |  | |
|  |  |  | *D. immitis* | fox | blood | 38 |  | |  | |  | |  | |  | |  | |  | |  | |  | |  | | 0 | |  | |
|  |  |  | *D. immitis* | dogs | blood | 97 |  | |  | |  | |  | |  | |  | |  | |  | |  | |  | | 1 | |  | |
|  |  |  | *D. repens* | dogs | blood | 97 |  | |  | |  | |  | |  | |  | |  | |  | |  | |  | | 1 | |  | |
|  |  |  | *D. immitis* | cat | blood | 207 |  | |  | |  | |  | |  | |  | |  | |  | |  | |  | | 0 | |  | |
|  |  |  | *D. repens* | jackal | blood | 55 |  | |  | |  | |  | |  | |  | |  | |  | |  | |  | | 0 | |  | |
|  |  |  | *D. repens* | fox | blood | 38 |  | |  | |  | |  | |  | |  | |  | |  | |  | |  | | 0 | |  | |
|  |  |  | *D. repens* | cat | blood | 207 |  | |  | |  | |  | |  | |  | |  | |  | |  | |  | | 0 | |  | |
| **(Pandey et al. 1987)** | Morocco | Rabat | *D. immitis* | dog | blood | 57 |  | |  | |  | | 7 | |  | |  | |  | |  | |  | |  | |  | |  | |
| **(Pasa et al. 2017)** | Türkiye | Aegean Coast | *D. immitis* | dog | blood | 46 |  | |  | |  | |  | |  | |  | |  | | 36 | |  | |  | |  | |  | |
| **(Raniel et al. 2006)** | Israel | None specified | *D. repens* | human | worms | 1 |  | |  | |  | |  | |  | | 1 | |  | |  | |  | |  | |  | |  | |
| **(Rjeibi et al. 2017)** | Tunisia | None specified | *D. immitis* | dog | blood | 200 |  | |  | |  | |  | |  | |  | |  | |  | |  | |  | | 29 | |  | |
|  |  |  | *D. repens* | dog | blood | 200 |  | |  | |  | |  | |  | |  | |  | |  | |  | |  | | 6 | |  | |
| **(Saied et al. 2011)** | Tunisia | None specified | *D. repens* | human | worms | 1 |  | |  | |  | |  | | 1 | |  | |  | |  | |  | |  | |  | |  | |
| **(Sari et al. 2013)** | Türkiye | Kulluk | *D. immitis* | dog | blood | 25 |  | |  | |  | |  | |  | |  | |  | | 14 | |  | |  | |  | |  | |
|  |  | Pirli | *D. immitis* | dog | blood | 26 |  | |  | |  | |  | |  | |  | |  | | 6 | |  | |  | |  | |  | |
|  |  | Baharli | *D. immitis* | dog | blood | 25 |  | |  | |  | |  | |  | |  | |  | | 16 | |  | |  | |  | |  | |
|  |  | Sogutlu | *D. immitis* | dog | blood | 24 |  | |  | |  | |  | |  | |  | |  | | 4 | |  | |  | |  | |  | |
| **(Sassi et al. 2006)** | Tunisia | Tunis | *D. repens* | human | worms | 1 |  | |  | |  | |  | | 1 | |  | |  | |  | |  | |  | |  | |  | |
| **(Selim et al. 2021)** | Egypt | Cairo | *D. immitis* | dog | serum | 230 |  | |  | |  | |  | |  | |  | |  | | 4 | |  | |  | |  | |  | |
|  |  | Giza | *D. immitis* | dog | serum | 110 |  | |  | |  | |  | |  | |  | |  | | 2 | |  | |  | |  | |  | |
|  |  | Al-Qalyubia | *D. immitis* | dog | serum | 60 |  | |  | |  | |  | |  | |  | |  | | 1 | |  | |  | |  | |  | |
|  |  | Al-Gharbia | *D. immitis* | dog | serum | 60 |  | |  | |  | |  | |  | |  | |  | | 0 | |  | |  | |  | |  | |
|  |  | Kafr El-Sheikh | *D. immitis* | dog | serum | 40 |  | |  | |  | |  | |  | |  | |  | | 0 | |  | |  | |  | |  | |
| **(Sevimli et al. 2007)** | Türkiye | unknown | *D. immitis* | dog | worms | 4 |  | |  | |  | |  | | 4 | |  | |  | |  | |  | |  | |  | |  | |
| **(Simsek and Ciftci 2016)** | Türkiye | Elazig | *D. immitis* | dog | blood | 161 |  | |  | |  | |  | |  | |  | |  | | 6 | |  | |  | | 3 | |  | |
|  |  |  | *D. repens* | dog | blood | 161 |  | |  | |  | |  | |  | |  | |  | |  | |  | |  | | 1 | |  | |
|  |  |  | *D. immitis + D. repens* co-infection | dog | blood | 161 |  | |  | |  | |  | |  | |  | |  | |  | |  | |  | | 3 | |  | |
| **(Simsek et al. 2011)** | Türkiye | Erzurum | *D. immitis* | dog | blood | 123 |  | |  | |  | | 6 | |  | |  | |  | |  | |  | |  | | 10 | |  | |
|  |  |  |  |  | serum | 93 |  | |  | |  | |  | |  | |  | |  | |  | | 2 | |  | |  | |  | |
| **(Simsek et al. 2008)** | Türkiye | Kocaeli | *D. immitis* | dog | blood | 71 |  | |  | |  | |  | |  | |  | |  | | 13 | |  | |  | | 0 | |  | |
|  |  | Sakarya | *D. immitis* | dog | blood | 65 |  | |  | |  | |  | |  | |  | |  | | 8 | |  | |  | | 0 | |  | |
|  |  | Elazig | *D. immitis* | dog | blood | 29 |  | |  | |  | |  | |  | |  | |  | | 0 | |  | |  | | 0 | |  | |
|  |  | Ankara | *D. immitis* | dog | blood | 27 |  | |  | |  | |  | |  | |  | |  | | 4 | |  | |  | | 0 | |  | |
|  |  | Mersin | *D. immitis* | dog | blood | 19 |  | |  | |  | |  | |  | |  | |  | | 2 | |  | |  | | 0 | |  | |
|  |  | Ankara | *D. immitis* | cat | blood | 15 |  | |  | |  | |  | |  | |  | |  | | N/A | |  | |  | | 0 | |  | |
| **(Soussi et al. 2004)** | Tunisia | None specified | *D. repens* | human | worms | 1 |  | |  | |  | |  | | 1 | |  | |  | |  | |  | |  | |  | |  | |
| **(Stayerman et al. 1999)** | Israel | None specified | *D. repens* | human | worms | 1 |  | |  | |  | |  | | 1 | |  | |  | |  | |  | |  | |  | |  | |
| **(Tahir et al. 2017)** | Algeria | None specified | *D. immitis* | dog | blood | 209 |  | |  | |  | |  | |  | |  | |  | |  | |  | |  | | 3 | |  | |
|  |  |  | *D. repens* | dog | blood | 209 |  | |  | |  | |  | |  | |  | |  | |  | |  | |  | | 0 | |  | |
| **(Tarello 2008)** | Kuwait | None specified | *D. immitis* | dog | blood | 381 | 0 | |  | |  | |  | |  | |  | |  | | 0 | |  | |  | |  | |  | |
|  |  | None specified | *D. repens* | dog | blood | 381 | 7 | |  | |  | |  | |  | |  | |  | |  | |  | |  | |  | |  | |
| **(Tarello 2002)** | United Arab Emirates | Dubai | *D. immitis* | dog | blood | 1 |  | |  | |  | |  | |  | |  | |  | |  | |  | |  | | 0 | |  | |
|  |  | Dubai | *D. repens* | dog | blood | 1 |  | |  | | 1 | | 0 | |  | |  | |  | |  | |  | |  | |  | |  | |
| **(Tarello 2003)** | Saudi Arabia | None specified | *D. repens* | dog | blood | 3 |  | |  | | 3 | | 0 | |  | |  | |  | |  | |  | |  | |  | |  | |
|  |  | None specified | *D. immitis* | dog | blood | 3 |  | |  | |  | |  | |  | |  | |  | | 1 | |  | |  | |  | |  | |
| **(Tarish et al. 1986)** | Iraq | Baghdad | *D. immitis* | dog | worms | 20 |  | |  | |  | |  | | 3 | |  | |  | |  | |  | |  | |  | |  | |
| **(Tasci and Kilic 2012)** | Türkiye | None specified | *D. immitis* | dog | blood | 240 |  | |  | |  | |  | |  | |  | | 52 | |  | |  | | 72 | | 60 | |  | |
| **(Ural et al. 2014)** | Türkiye | None specified | *D. immitis* | dog | blood | 307 |  | |  | |  | |  | |  | |  | |  | | 11 | |  | |  | |  | |  | |
| **(Voyvoda et al. 2004)** | Türkiye | Aydin | *D. immitis* | dog | blood | 158 | 22 | |  | |  | |  | |  | |  | |  | |  | |  | |  | |  | |  | |
| **(Yaman et al. 2009)** | Türkiye | Shoreline area (Iskenderun) | *D. immitis* | dog | blood | 88 | 9 | |  | |  | |  | |  | |  | |  | | 23 | |  | |  | |  | |  | |
|  |  | Riverside (Antakya) | *D. immitis* | dog | blood | 55 | 5 | |  | |  | |  | |  | |  | |  | | 15 | |  | |  | |  | |  | |
|  |  | Lowland (Kirikhan and Hassa) | *D. immitis* | dog | blood | 47 | 6 | |  | |  | |  | |  | |  | |  | | 12 | |  | |  | |  | |  | |
|  |  | Mountanious (Yayladagi and Altinozu) | *D. immitis* | dog | blood | 79 | 5 | |  | |  | |  | |  | |  | |  | | 11 | |  | |  | |  | |  | |
| **(Yildirim et al. 2007)** | Türkiye | Kayseri | *D. immitis* | dog | blood | 140 |  | |  | |  | |  | |  | |  | | 17 | | 21 | |  | |  | |  | |  | |
|  |  | Incesu | *D. immitis* | dog | blood | 34 |  | |  | |  | |  | |  | |  | | 0 | | 1 | |  | |  | |  | |  | |
|  |  | Pinarbasi | *D. immitis* | dog | blood | 18 |  | |  | |  | |  | |  | |  | | 0 | | 0 | |  | |  | |  | |  | |
|  |  | Felahiye | *D. immitis* | dog | blood | 18 |  | |  | |  | |  | |  | |  | | 1 | | 0 | |  | |  | |  | |  | |
|  |  | Talas | *D. immitis* | dog | blood | 32 |  | |  | |  | |  | |  | |  | | 1 | | 1 | |  | |  | |  | |  | |
|  |  | Tomarza | *D. immitis* | dog | blood | 17 |  | |  | |  | |  | |  | |  | | 0 | | 0 | |  | |  | |  | |  | |
|  |  | Yahyali | *D. immitis* | dog | blood | 21 |  | |  | |  | |  | |  | |  | | 0 | | 1 | |  | |  | |  | |  | |
| **(Yildirim et al. 2011)** | Türkiye | Kayseri | *D. immitis* | *Aedes vexans* head-thorax pools |  | 3,179 individual mosquitoes, both sampling years. Number of pools not provided |  | |  | |  | |  | |  | |  | |  | |  | |  | |  | | 3 positive pools, head-thorax only | |  | |
|  |  |  | *D. immitis* | *Aedes vexans* abdomen pools |  |  |  | |  | |  | |  | |  | |  | |  | |  | |  | |  | | 1 positive pool, abdomen only | |  | |
|  |  |  | *D. immitis* | *Culex pipiens* head-thorax pools |  | 2,589 individual mosquitoes, both sampling years. Number of pools not provided |  | |  | |  | |  | |  | |  | |  | |  | |  | |  | |  | |  | |
|  |  |  | *D. immitis* | *Culex pipiens* abdomen pools |  |  |  | |  | |  | |  | |  | |  | |  | |  | |  | |  | |  | |  | |
|  |  |  | *D. immitis* | *Culex theileri* head-thorax pools |  | 193 individual mosquitoes, both sampling years. Number of pools not provided |  | |  | |  | |  | |  | |  | |  | |  | |  | |  | | 0 positive pools | |  | |
|  |  |  | *D. immitis* | *Culex theileri* abdomen pools |  |  |  | |  | |  | |  | |  | |  | |  | |  | |  | |  | | 0 positive pools | |  | |
|  |  |  | *D. immitis* | *Culex hortensis* head-thorax pools |  | 36 individual mosquitoes, both sampling years. Number of pools not provided |  | |  | |  | |  | |  | |  | |  | |  | |  | |  | | 0 positive pools | |  | |
|  |  |  | *D. immitis* | *Culex hortensis* abdomen pools |  |  |  | |  | |  | |  | |  | |  | |  | |  | |  | |  | | 0 positive pools | |  | |
|  |  |  | *D. immitis* | *Culiseta annulata* head-thorax pools |  | 94 individual mosquitoes only in 2009. Number of pools not provided |  | |  | |  | |  | |  | |  | |  | |  | |  | |  | | 0 positive pools | |  | |
|  |  |  | *D. immitis* | *Culiseta annulata* abdomen pools |  |  |  | |  | |  | |  | |  | |  | |  | |  | |  | |  | | 0 positive pools | |  | |
|  |  |  | *D. immitis* | *Anopheles maculipennis* head-thorax pools |  | 62 individual mosquitoes, only in 2009. Number of pools not provided |  | |  | |  | |  | |  | |  | |  | |  | |  | |  | | 0 positive pools | |  | |
|  |  |  | *D. immitis* | *Anopheles maculipennis* abdomen pools |  |  |  | |  | |  | |  | |  | |  | |  | |  | |  | |  | | 0 positive pools | |  | |
| **(Yildiz et al. 2008)** | Türkiye | Kirklareli | *D. immitis* | dog | blood | 172 | 10 | |  | |  | |  | |  | |  | |  | |  | |  | |  | |  | |  | |
|  |  |  | *D. immitis* | dog | blood | 142 |  | |  | |  | |  | |  | |  | |  | | 38 | |  | |  | |  | |  | |
| **(Ziadi et al. 2005)** | Tunisia | None specified | *D. immitis* | human | worms | 1 |  | |  | |  | |  | | 1 | |  | |  | |  | |  | |  | |  | |  | |
|  |  |  | *D. repens* | human | worms | 1 |  |  | |  | |  | | 1 | |  | |  | |  | |  | |  | |  | |  | |  |
