# Supplementary material for: A meta-analysis on Dirofilaria immitis and Dirofilaria repens in countries of North Africa and the Middle East
Source: Parasitology. 2025 Apr 1;152(4):347–65. doi: 10.1017/S003118202500037X (PMC12186096; doi:10.1017/S003118202500037X)
Supplement: Izenour et al. supplementary material 4 — Izenour et al. supplementary material [file S003118202500037Xsup004.docx]

**Supplement 3**

**Table of publications and observations included in the meta-analysis model, ‘meta-analysis dataset’**

| **record**  **ID** | **publication**  **ID** | **publication (last name year)** | **year** | **total sample** | **number positive** | **diagnostic method** | **diagnostic sample** | **host species** | ***Dirofilaria* spp.** | **country** | **continent** |
| --- | --- | --- | --- | --- | --- | --- | --- | --- | --- | --- | --- |
| 1 | 2 | (Abdullah et al. 2021) | 2021 | 203 | 2 | PCR | blood | dogs | *D. repens* | Egypt | Africa |
| 2 | 4 | (Adanir et al. 2013) | 2013 | 142 | 31 | ELISA (DiroCHEK^®^) | serum | dogs | *D. immitis* | Türkiye | Asia |
| 3 | 5 | (Al-Kappany et al. 2011) | 2018 | 174 | 6 | SNAP Feline Triple | serum | cat | *D. immitis* | Egypt | Africa |
| 4 | 8 | (Atas et al. 2018) | 2018 | 306 | 9 | ELISA (DiroCHEK^®^) | blood | dogs | *D. immitis* | Türkiye | Asia |
| 5 | 8 | (Atas et al. 2018) | 2016 | 306 | 9 | PCR | blood | dogs | *D. immitis* | Türkiye | Asia |
| 6 | 8 | (Atas et al. 2018) | 2016 | 306 | 4 | Modified Knotts | blood | dogs | *D. immitis* | Türkiye | Asia |
| 7 | 17 | (Cetinkaya et al. 2016) | 2016 | 100 | 0 | ELISA (SNAP^®^ 3Dx^®^) | blood | dogs | *D. immitis* | Türkiye | Asia |
| 8 | 17 | (Cetinkaya et al. 2016) | 2016 | 102 | 15 | ELISA (SNAP^®^ 3Dx^®^) | blood | dogs | *D. immitis* | Türkiye | Asia |
| 9 | 17 | (Cetinkaya et al. 2016) | 2016 | 100 | 1 | ELISA (SNAP^®^ 3Dx^®^) | blood | dogs | *D. immitis* | Türkiye | Asia |
| 10 | 17 | (Cetinkaya et al. 2016) | 2016 | 100 | 11 | ELISA (SNAP^®^ 3Dx^®^) | blood | dogs | *D. immitis* | Türkiye | Asia |
| 11 | 17 | (Cetinkaya et al. 2016) | 2016 | 100 | 0 | PCR | blood | dogs | *D. immitis* | Türkiye | Asia |
| 12 | 17 | (Cetinkaya et al. 2016) | 2016 | 102 | 5 | PCR | blood | dogs | *D. immitis* | Türkiye | Asia |
| 13 | 17 | (Cetinkaya et al. 2016) | 2016 | 100 | 0 | PCR | blood | dogs | *D. immitis* | Türkiye | Asia |
| 14 | 17 | (Cetinkaya et al. 2016) | 2016 | 100 | 6 | PCR | blood | dogs | *D. immitis* | Türkiye | Asia |
| 15 | 17 | (Cetinkaya et al. 2016) | 2016 | 100 | 0 | Microscopy (Giemsa stain) | blood | dogs | *D. immitis* | Türkiye | Asia |
| 16 | 17 | (Cetinkaya et al. 2016) | 2016 | 102 | 0 | Microscopy (Giemsa stain) | blood | dogs | *D. immitis* | Türkiye | Asia |
| 17 | 17 | (Cetinkaya et al. 2016) | 2021 | 100 | 0 | Microscopy (Giemsa stain) | blood | dogs | *D. immitis* | Türkiye | Asia |
| 18 | 17 | (Cetinkaya et al. 2016) | 2021 | 100 | 3 | Microscopy (Giemsa stain) | blood | dogs | *D. immitis* | Türkiye | Asia |
| 19 | 19 | (Ceylan et al. 2021) | 2021 | 31 | 0 | Antigen Rapid CaniV-4 (Leish) Test Kit, BioNote Co. | blood | dogs | *D. immitis* | Türkiye | Asia |
| 20 | 19 | (Ceylan et al. 2021) | 2021 | 7 | 0 | Antigen Rapid CaniV-4 (Leish) Test Kit, BioNote Co. | blood | dogs | *D. immitis* | Türkiye | Asia |
| 21 | 19 | (Ceylan et al. 2021) | 2021 | 13 | 0 | Antigen Rapid CaniV-4 (Leish) Test Kit, BioNote Co. | blood | dogs | *D. immitis* | Türkiye | Asia |
| 22 | 19 | Ceylan (Ceylan et al. 2021) | 2021 | 26 | 1 | Antigen Rapid CaniV-4 (Leish) Test Kit, BioNote Co. | blood | dogs | *D. immitis* | Türkiye | Asia |
| 23 | 19 | (Ceylan et al. 2021) | 2021 | 11 | 1 | Antigen Rapid CaniV-4 (Leish) Test Kit, BioNote Co. | blood | dogs | *D. immitis* | Türkiye | Asia |
| 24 | 19 | (Ceylan et al. 2021) | 2021 | 7 | 0 | Antigen Rapid CaniV-4 (Leish) Test Kit, BioNote Co. | blood | dogs | *D. immitis* | Türkiye | Asia |
| 25 | 19 | (Ceylan et al. 2021) | 2021 | 10 | 0 | Antigen Rapid CaniV-4 (Leish) Test Kit, BioNote Co. | blood | dogs | *D. immitis* | Türkiye | Asia |
| 26 | 19 | (Ceylan et al. 2021) | 2021 | 25 | 0 | Antigen Rapid CaniV-4 (Leish) Test Kit, BioNote Co. | blood | dogs | *D. immitis* | Türkiye | Asia |
| 27 | 19 | (Ceylan et al. 2021) | 2021 | 12 | 0 | Antigen Rapid CaniV-4 (Leish) Test Kit, BioNote Co. | blood | dogs | *D. immitis* | Türkiye | Asia |
| 28 | 19 | (Ceylan et al. 2021) | 2021 | 36 | 1 | Antigen Rapid CaniV-4 (Leish) Test Kit, BioNote Co. | blood | dogs | *D. immitis* | Türkiye | Asia |
| 29 | 19 | (Ceylan et al. 2021) | 2017 | 29 | 0 | Antigen Rapid CaniV-4 (Leish) Test Kit, BioNote Co. | blood | dogs | *D. immitis* | Türkiye | Asia |
| 30 | 19 | (Ceylan et al. 2021) | 2017 | 41 | 0 | Antigen Rapid CaniV-4 (Leish) Test Kit, BioNote Co. | blood | dogs | *D. immitis* | Türkiye | Asia |
| 31 | 39 | (Elhamiani Khatat et al. 2017) | 2017 | 5 | 1 | Elisa (SNAP® 4Dx® Plus) | blood | dogs | *D. immitis* | Morocco | Africa |
| 32 | 39 | (Elhamiani Khatat et al. 2017) | 2017 | 4 | 0 | Elisa (SNAP® 4Dx® Plus) | blood | dogs | *D. immitis* | Morocco | Africa |
| 33 | 39 | (Elhamiani Khatat et al. 2017) | 2017 | 78 | 28 | Elisa (SNAP® 4Dx® Plus) | blood | dogs | *D. immitis* | Morocco | Africa |
| 34 | 39 | (Elhamiani Khatat et al. 2017) | 2017 | 57 | 1 | Elisa (SNAP® 4Dx® Plus) | blood | dogs | *D. immitis* | Morocco | Africa |
| 35 | 39 | (Elhamiani Khatat et al. 2017) | 2017 | 25 | 5 | Elisa (SNAP® 4Dx® Plus) | blood | dogs | *D. immitis* | Morocco | Africa |
| 36 | 39 | (Elhamiani Khatat et al. 2017) | 2017 | 32 | 0 | Elisa (SNAP® 4Dx® Plus) | blood | dogs | *D. immitis* | Morocco | Africa |
| 37 | 39 | (Elhamiani Khatat et al. 2017) | 2017 | 16 | 0 | Elisa (SNAP® 4Dx® Plus) | blood | dogs | *D. immitis* | Morocco | Africa |
| 38 | 46 | (Guven et al. 2017) | 2011 | 133 | 2 | PCR | blood | dogs | *D. immitis* | Türkiye | Asia |
| 39 | 46 | (Guven et al. 2017) | 2022 | 133 | 0 | PCR | blood | dogs | *D. repens* | Türkiye | Asia |
| 40 | 51 | (Icen et al. 2011) | 2022 | 82 | 2 | ELISA (SNAP^®^ 3Dx^®^) | blood | dogs | *D. immitis* | Türkiye | Asia |
| 41 | 130 | (Izenour et al. 2022) | 2022 | 114 | 0 | Elisa (SNAP® 4Dx® Plus) | blood | dogs | *D. immitis* | Egypt | Africa |
| 42 | 130 | (Izenour et al. 2022) | 2022 | 114 | 0 | PCR | blood | dogs | *D. immitis* | Egypt | Africa |
| 43 | 130 | (Izenour et al. 2022) | 2022 | 114 | 0 | PCR | blood | dogs | *D. repens* | Egypt | Africa |
| 44 | 60 | (Köse and Erdogan 2012) | 2012 | 19 | 0 | ELISA (DiroCHEK^®^) | blood | dogs | *D. immitis* | Türkiye | Asia |
| 45 | 60 | (Köse and Erdogan 2012) | 2012 | 33 | 5 | ELISA (DiroCHEK^®^) | blood | dogs | *D. immitis* | Türkiye | Asia |
| 46 | 60 | (Köse and Erdogan 2012) | 2012 | 34 | 6 | ELISA (DiroCHEK^®^) | blood | dogs | *D. immitis* | Türkiye | Asia |
| 47 | 60 | (Köse and Erdogan 2012) | 2012 | 51 | 1 | ELISA (DiroCHEK^®^) | blood | dogs | *D. immitis* | Türkiye | Asia |
| 48 | 60 | (Köse and Erdogan 2012) | 2012 | 28 | 4 | ELISA (DiroCHEK^®^) | blood | dogs | *D. immitis* | Türkiye | Asia |
| 49 | 60 | (Köse and Erdogan 2012) | 2012 | 25 | 3 | ELISA (DiroCHEK^®^) | blood | dogs | *D. immitis* | Türkiye | Asia |
| 50 | 60 | (Köse and Erdogan 2012) | 2012 | 25 | 1 | ELISA (DiroCHEK^®^) | blood | dogs | *D. immitis* | Türkiye | Asia |
| 51 | 60 | (Köse and Erdogan 2012) | 2012 | 42 | 4 | ELISA (DiroCHEK^®^) | blood | dogs | *D. immitis* | Türkiye | Asia |
| 52 | 60 | (Köse and Erdogan 2012) | 2012 | 23 | 2 | ELISA (DiroCHEK^®^) | blood | dogs | *D. immitis* | Türkiye | Asia |
| 53 | 60 | (Köse and Erdogan 2012) | 2012 | 37 | 1 | ELISA (DiroCHEK^®^) | blood | dogs | *D. immitis* | Türkiye | Asia |
| 54 | 61 | (Kozan et al. 2007) | 2009 | 137 | 5 | Modified Knotts | blood | dogs | *D. immitis* | Türkiye | Asia |
| 55 | 61 | (Kozan et al. 2007) | 2009 | 146 | 2 | Modified Knotts | blood | dogs | *D. immitis* | Türkiye | Asia |
| 56 | 61 | (Kozan et al. 2007) | 2018 | 137 | 0 | Microscopy blood smear | blood | dogs | *D. immitis* | Türkiye | Asia |
| 57 | 61 | (Kozan et al. 2007) | 2003 | 146 | 0 | Microscopy blood smear | blood | dogs | *D. immitis* | Türkiye | Asia |
| 129 | 68 | (Mazaki-Tovi et al. 2016) | 2016 | 4 | 3 | Microscopy blood smear | blood | dogs | *D. repens* | Israel | Asia |
| 130 | 68 | (Mazaki-Tovi et al. 2016) | 2016 | 4 | 4 | PCR | blood | dogs | *D. repens* | Israel | Asia |
| 58 | 71 | (Meriem-Hind and Mohamed 2009) | 2003 | 184 | 45 | ELISA (PetChek^®^) | blood | dogs | *D. immitis* | Algeria | Africa |
| 59 | 71 | (Meriem-Hind and Mohamed 2009) | 2018 | 184 | 34 | Modified Knotts | blood | dogs | *D. immitis* | Algeria | Africa |
| 123 | 81 | (Obaidat and Alshehabat 2018) | 2018 | 161 | 0 | Elisa (SNAP® 4Dx® Plus) | serum | dogs | *D. immitis* | Jordan | Asia |
| 60 | 82 | (Oge et al. 2003) | 2018 | 280 | 24 | ELISA (PetChek^®^) | blood | dogs | *D. immitis* | Türkiye | Asia |
| 61 | 82 | (Oge et al. 2003) | 2018 | 280 | 2 | Membrane Filtration-Acid Phosphate Histochemical Staining | blood | dogs | *D. immitis* | Türkiye | Asia |
| 129 | 83 | (Omar et al. 2018) | 2005 | 294 | 23 | ELISA (DiroCHEK^®^) | serum | dogs | *D. immitis* | Saudi Arabia | Asia |
| 130 | 83 | (Omar et al. 2018) | 2005 | 190 | 3 | ELISA (DiroCHEK^®^) | serum | cat | *D. immitis* | Saudi Arabia | Asia |
| 62 | 84 | (Oncel and Vural 2005) | 2019 | 117 | 0 | ELISA (PetChek^®^) | blood | dogs | *D. immitis* | Türkiye | Asia |
| 63 | 84 | (Oncel and Vural 2005) | 2019 | 263 | 4 | ELISA (PetChek^®^) | blood | dogs | *D. immitis* | Türkiye | Asia |
| 119 | 85 | (Otranto et al. 2019) | 2019 | 97 | 1 | PCR | blood | dogs | *D. immitis* | Iraq | Asia |
| 120 | 85 | (Otranto et al. 2019) | 2019 | 97 | 1 | PCR | blood | dogs | *D. repens* | Iraq | Asia |
| 121 | 85 | (Otranto et al. 2019) | 2019 | 207 | 0 | PCR | blood | cat | *D. immitis* | Iraq | Asia |
| 122 | 85 | (Otranto et al. 2019) | 2019 | 207 | 0 | PCR | blood | cat | *D. repens* | Iraq | Asia |
| 64 | 87 | (Pandey et al. 1987) | 2013 | 57 | 7 | Microscopy (Giemsa stain) | blood | dogs | *D. immitis* | Morocco | Africa |
| 65 | 88 | (Pasa et al. 2017) | 2013 | 46 | 36 | Elisa (SNAP® 4Dx® Plus) | blood | dogs | *D. immitis* | Türkiye | Asia |
| 124 | 92 | (Rjeibi et al. 2017) | 2013 | 200 | 29 | PCR | blood | dogs | *D. immitis* | Tunisia | Africa |
| 125 | 92 | (Rjeibi et al. 2017) | 2013 | 200 | 6 | PCR | blood | dogs | *D. repens* | Tunisia | Africa |
| 66 | 97 | (Sari et al. 2013) | 2021 | 25 | 14 | ELISA (SNAP^®^ 3Dx^®^) | blood | dogs | *D. immitis* | Türkiye | Asia |
| 67 | 97 | (Sari et al. 2013) | 2021 | 26 | 6 | ELISA (SNAP^®^ 3Dx^®^) | blood | dogs | *D. immitis* | Türkiye | Asia |
| 68 | 97 | (Sari et al. 2013) | 2021 | 25 | 16 | ELISA (SNAP^®^ 3Dx^®^) | blood | dogs | *D. immitis* | Türkiye | Asia |
| 69 | 97 | (Sari et al. 2013) | 2021 | 24 | 4 | ELISA (SNAP^®^ 3Dx^®^) | blood | dogs | *D. immitis* | Türkiye | Asia |
| 70 | 104 | (Selim et al. 2021) | 2021 | 230 | 4 | Elisa (SNAP® 4Dx® Plus) | serum | dogs | *D. immitis* | Egypt | Africa |
| 71 | 104 | (Selim et al. 2021) | 2016 | 110 | 2 | Elisa (SNAP® 4Dx® Plus) | serum | dogs | *D. immitis* | Egypt | Africa |
| 72 | 104 | (Selim et al. 2021) | 2016 | 60 | 1 | Elisa (SNAP® 4Dx® Plus) | serum | dogs | *D. immitis* | Egypt | Africa |
| 73 | 104 | (Selim et al. 2021) | 2016 | 60 | 0 | Elisa (SNAP® 4Dx® Plus) | serum | dogs | *D. immitis* | Egypt | Africa |
| 74 | 104 | (Selim et al. 2021) | 2016 | 40 | 0 | Elisa (SNAP® 4Dx® Plus) | serum | dogs | *D. immitis* | Egypt | Africa |
| 75 | 106 | (Simsek and Ciftci 2016) | 2016 | 161 | 6 | ELISA (Filarcheck) | blood | dogs | *D. immitis* | Türkiye | Asia |
| 76 | 106 | (Simsek and Ciftci 2016) | 2016 | 161 | 1 | PCR | blood | dogs | *D. repens* | Türkiye | Asia |
| 77 | 106 | (Simsek and Ciftci 2016) | 2016 | 161 | 3 | PCR | blood | dogs | *D. immitis* | Türkiye | Asia |
| 78 | 107 | (Simsek et al. 2011) | 2011 | 123 | 10 | PCR | blood | dogs | *D. immitis* | Türkiye | Asia |
| 79 | 107 | (Simsek et al. 2011) | 2011 | 123 | 6 | Microscopy blood smear | blood | dogs | *D. immitis* | Türkiye | Asia |
| 80 | 108 | (Simsek et al. 2008) | 2008 | 71 | 13 | ELISA (DiroCHEK^®^) | serum | dogs | *D. immitis* | Türkiye | Asia |
| 81 | 108 | (Simsek et al. 2008) | 2008 | 65 | 8 | ELISA (DiroCHEK^®^) | serum | dogs | *D. immitis* | Türkiye | Asia |
| 82 | 108 | (Simsek et al. 2008) | 2008 | 29 | 0 | ELISA (DiroCHEK^®^) | serum | dogs | *D. immitis* | Türkiye | Asia |
| 83 | 108 | (Simsek et al. 2008) | 2008 | 27 | 4 | ELISA (DiroCHEK^®^) | serum | dogs | *D. immitis* | Türkiye | Asia |
| 84 | 108 | (Simsek et al. 2008) | 2008 | 19 | 2 | ELISA (DiroCHEK^®^) | serum | dogs | *D. immitis* | Türkiye | Asia |
| 85 | 108 | (Simsek et al. 2008) | 2008 | 71 | 0 | PCR | blood | dogs | *D. immitis* | Türkiye | Asia |
| 86 | 108 | (Simsek et al. 2008) | 2008 | 65 | 0 | PCR | blood | dogs | *D. immitis* | Türkiye | Asia |
| 87 | 108 | (Simsek et al. 2008) | 2008 | 29 | 0 | PCR | blood | dogs | *D. immitis* | Türkiye | Asia |
| 88 | 108 | (Simsek et al. 2008) | 2008 | 27 | 0 | PCR | blood | dogs | *D. immitis* | Türkiye | Asia |
| 89 | 108 | (Simsek et al. 2008) | 2008 | 19 | 0 | PCR | blood | dogs | *D. immitis* | Türkiye | Asia |
| 90 | 108 | (Simsek et al. 2008) | 2008 | 15 | 0 | PCR | blood | cat | *D. immitis* | Türkiye | Asia |
| 91 | 114 | (Tahir et al. 2017) | 2017 | 209 | 3 | PCR | blood | dogs | *D. immitis* | Algeria | Africa |
| 92 | 114 | (Tahir et al. 2017) | 2017 | 209 | 0 | PCR | blood | dogs | *D. repens* | Algeria | Africa |
| 126 | 115 | (Tarello 2008) | 2008 | 381 | 0 | ELISA (DiroCHEK^®^) | blood | dogs | *D. immitis* | Kuwait | Asia |
| 127 | 115 | (Tarello 2008) | 2008 | 381 | 7 | Knotts | blood | dogs | *D. repens* | Kuwait | Asia |
| 128 | 115 | (Tarello 2008) | 2008 | 381 | 0 | Knotts | blood | dogs | *D. immitis* | Kuwait | Asia |
| 93 | 120 | (Tasci and Kilic 2012) | 2012 | 240 | 60 | PCR | blood | dogs | *D. immitis* | Türkiye | Asia |
| 94 | 120 | (Tasci and Kilic 2012) | 2012 | 240 | 52 | Membrane Filtration-Acid Phosphate Histochemical Staining | blood | dogs | *D. immitis* | Türkiye | Asia |
| 95 | 123 | (Ural et al. 2014) | 2014 | 307 | 11 | ELISA SNAP^®^ 4Dx^®^ | blood | dogs | *D. immitis* | Türkiye | Asia |
| 96 | 124 | (Voyvoda et al. 2004) | 2004 | 158 | 22 | Modified Knotts | blood | dogs | *D. immitis* | Türkiye | Asia |
| 97 | 125 | (Yaman et al. 2009) | 2009 | 88 | 23 | ELISA (DiroCHEK^®^) | blood | dogs | *D. immitis* | Türkiye | Asia |
| 98 | 125 | (Yaman et al. 2009) | 2009 | 55 | 15 | ELISA (DiroCHEK^®^) | blood | dogs | *D. immitis* | Türkiye | Asia |
| 99 | 125 | (Yaman et al. 2009) | 2009 | 47 | 12 | ELISA (DiroCHEK^®^) | blood | dogs | *D. immitis* | Türkiye | Asia |
| 100 | 125 | (Yaman et al. 2009) | 2009 | 79 | 11 | ELISA (DiroCHEK^®^) | blood | dogs | *D. immitis* | Türkiye | Asia |
| 101 | 125 | (Yaman et al. 2009) | 2009 | 88 | 9 | Modified Knotts | blood | dogs | *D. immitis* | Türkiye | Asia |
| 102 | 125 | (Yaman et al. 2009) | 2009 | 55 | 5 | Modified Knotts | blood | dogs | *D. immitis* | Türkiye | Asia |
| 103 | 125 | (Yaman et al. 2009) | 2009 | 47 | 6 | Modified Knotts | blood | dogs | *D. immitis* | Türkiye | Asia |
| 104 | 125 | (Yaman et al. 2009) | 2009 | 79 | 5 | Modified Knotts | blood | dogs | *D. immitis* | Türkiye | Asia |
| 105 | 126 | (Yildirim et al. 2007) | 2007 | 140 | 21 | ELISA (DiroCHEK^®^) | blood | dogs | *D. immitis* | Türkiye | Asia |
| 106 | 126 | (Yildirim et al. 2007) | 2007 | 34 | 1 | ELISA (DiroCHEK^®^) | blood | dogs | *D. immitis* | Türkiye | Asia |
| 107 | 126 | (Yildirim et al. 2007) | 2007 | 18 | 0 | ELISA (DiroCHEK^®^) | blood | dogs | *D. immitis* | Türkiye | Asia |
| 108 | 126 | (Yildirim et al. 2007) | 2007 | 18 | 0 | ELISA (DiroCHEK^®^) | blood | dogs | *D. immitis* | Türkiye | Asia |
| 109 | 126 | (Yildirim et al. 2007) | 2007 | 32 | 1 | ELISA (DiroCHEK^®^) | blood | dogs | *D. immitis* | Türkiye | Asia |
| 110 | 126 | (Yildirim et al. 2007) | 2007 | 17 | 0 | ELISA (DiroCHEK^®^) | blood | dogs | *D. immitis* | Türkiye | Asia |
| 111 | 126 | (Yildirim et al. 2007) | 2007 | 21 | 1 | ELISA (DiroCHEK^®^) | blood | dogs | *D. immitis* | Türkiye | Asia |
| 112 | 126 | (Yildirim et al. 2007) | 2007 | 140 | 17 | Membrane Filtration-Acid Phosphate Histochemical Staining | blood | dogs | *D. immitis* | Türkiye | Asia |
| 113 | 126 | (Yildirim et al. 2007) | 2007 | 34 | 0 | Membrane Filtration-Acid Phosphate Histochemical Staining | blood | dogs | *D. immitis* | Türkiye | Asia |
| 114 | 126 | (Yildirim et al. 2007) | 2007 | 18 | 0 | Membrane Filtration-Acid Phosphate Histochemical Staining | blood | dogs | *D. immitis* | Türkiye | Asia |
| 115 | 126 | (Yildirim et al. 2007) | 2007 | 18 | 1 | Membrane Filtration-Acid Phosphate Histochemical Staining | blood | dogs | *D. immitis* | Türkiye | Asia |
| 116 | 126 | (Yildirim et al. 2007) | 2007 | 32 | 1 | Membrane Filtration-Acid Phosphate Histochemical Staining | blood | dogs | *D. immitis* | Türkiye | Asia |
| 117 | 126 | (Yildirim et al. 2007) | 2007 | 17 | 0 | Membrane Filtration-Acid Phosphate Histochemical Staining | blood | dogs | *D. immitis* | Türkiye | Asia |
| 118 | 126 | (Yildirim et al. 2007) | 2007 | 21 | 0 | Membrane Filtration-Acid Phosphate Histochemical Staining | blood | dogs | *D. immitis* | Türkiye | Asia |
