# Supplementary material for: A meta-analysis on Dirofilaria immitis and Dirofilaria repens in countries of North Africa and the Middle East
Source: Parasitology. 2025 Apr 1;152(4):347–65. doi: 10.1017/S003118202500037X (PMC12186096; doi:10.1017/S003118202500037X)
Supplement: Izenour et al. supplementary material 5 — Izenour et al. supplementary material [file S003118202500037Xsup005.docx]

**Supplement 5 – Moderator Analysis Output**

**Supplement 5A - Moderator analysis by continent of origin**

| **Continent** | **k events** | **proportion % (95% CI)** | **I^2^ %** |
| --- | --- | --- | --- |
| Asia | 108 | 2.5 (1.6 – 3.9) | 81.0 |
| Africa | 24 | 2.0 (0.7– 5.2) | 84.4 |
| Between group p-value: 0.66 | | | |

**Supplement 5B -****Moderator analysis by host species**

| **Host Species** | **k events** | **proportion % (95% CI)** | **I^2^ %** |
| --- | --- | --- | --- |
| Dog | 127 | 2.56 (1.7 – 3.9) | 81.7 |
| Cat | 5 | 0.48 (0.02 – 11.5) | 0.0 |
| Between group p-value: 0.16 | | | |

**Supplement 5c - Moderator analysis by *Dirofilaria* species**

| ***Dirofilaria* species** | **k events** | **proportion % (95% CI)** | **I^2^ %** |
| --- | --- | --- | --- |
| *D. immitis* | 121 | 2.7 (1.8 – 4.0) | 81.0 |
| *D. repens* | 11 | 0.9 (0.05 – 14.3) | 55.1 |
| Between group p-value: 0.39 | | | |

**Supplement 5D - Moderator analysis by diagnostic test method**

| **Diagnostic method** | **k events** | **proportion % (95% CI)** | **I^2^ %** |
| --- | --- | --- | --- |
| Antigen Rapid CaniV-4 (Leish) Test Kit, BioNote Co. | 12 | 1.2 (0.3 – 4.2) | 0.0 |
| ELISA (SNAP^®^ 4Dx^®^ Plus) | 15 | 1.3 (0.2 – 8.9) | 88.0 |
| ELISA (DiroCHEK^®^) | 31 | 6.5 (4.0 – 10.5) | 69.5 |
| ELISA (FilarCHECK) | 1 | 3.7 (1.7 – 8.1) | -- |
| ELISA (PetChek^®^) | 4 | 3.4 (0.1 – 46.1) | 93.3 |
| ELISA (SNAP^®^ 3Dx^®^) | 9 | 10.1 (2.3 – 35.1) | 87.9 |
| ELISA (SNAP^®^ 4Dx ^®^) | 1 | 3.6 (2.0 – 6.4) | -- |
| Knotts | 2 | 0.3 (0.0 – 100.0) | 0.0 |
| Membrane Filtration-Acid Phosphate Histochemical Staining | 9 | 2.2 (0.4 – 11.2) | 75.7 |
| Microscopy (Giemsa stain) | 5 | 0.5 (0.0 – 33.9) | 11.1 |
| Microscopy blood smear | 4 | 0.8 (0.0 – 97.9) | 72.7 |
| Modified Knotts | 9 | 6.4 (3.1 – 12.8) | 83.8 |
| PCR | 29 | 0.7 (0.2 – 2.3) | 81.1 |
| SNAP^®^ Feline Triple^®^ | 1 | 3.5 (1.6 – 7.5) | -- |
| Between group p-value: 0.0084 | | | |

**Supplement 5E- Moderator analysis by aggregated diagnostic test type**

| **Original ‘diagnostic method’** | **Aggregate** | **k events** | **proportion % (95% CI)** | **I^2^ %** |
| --- | --- | --- | --- | --- |
| Antigen Rapid CaniV-4 (Leish) Test Kit, BioNote Co. | Rapid Test (antigen) | 74 | 3.9 (2.4 – 6.3) | 82.3 |
| ELISA (SNAP^®^ 4Dx^®^ Plus) |  |  |  |  |
| ELISA (DiroCHEK^®^) |  |  |  |  |
| ELISA (FilarCHECK) |  |  |  |  |
| ELISA (PetChek^®^) |  |  |  |  |
| ELISA (SNAP^®^ 3Dx^®^) |  |  |  |  |
| ELISA (SNAP^®^ 4Dx ^®^) |  |  |  |  |
| SNAP^®^ Feline Triple^®^ |  |  |  |  |
| Knotts | Microscopy | 29 | 2.3 (0.98 – 5.2) | 79.5 |
| Membrane Filtration-Acid Phosphate Histochemical Staining |  |  |  |  |
| Microscopy (Giemsa stain) |  |  |  |  |
| Microscopy blood smear |  |  |  |  |
| Modified Knotts |  |  |  |  |
| PCR | PCR | 29 | 0.7 (0.2 – 2.4) | 81.1 |
| Between group p-value: 0.0213 | | | | |
